# Mycobacterial Infection of Precision-Cut Lung Slices Reveals Type 1 Interferon Pathway Is Locally Induced by *Mycobacterium bovis* but Not *M. tuberculosis* in a Cattle Breed

**DOI:** 10.3389/fvets.2021.696525

**Published:** 2021-07-09

**Authors:** Aude Remot, Florence Carreras, Anthony Coupé, Émilie Doz-Deblauwe, Maria L. Boschiroli, John A. Browne, Quentin Marquant, Delphyne Descamps, Fabienne Archer, Abraham Aseffa, Pierre Germon, Stephen V. Gordon, Nathalie Winter

**Affiliations:** ^1^INRAE, Université de Tours, Nouzilly, France; ^2^Paris-Est University, National Reference Laboratory for Tuberculosis, Animal Health Laboratory, Anses, Maisons-Alfort, France; ^3^UCD School of Agriculture and Food Science, University College Dublin, Dublin, Ireland; ^4^INRAE, Université Paris-Saclay, UVSQ, Jouy-en-Josas, France; ^5^INRAE, UMR754, Viral Infections and Comparative Pathology, IVPC, Univ Lyon, Université Claude Bernard Lyon 1, EPHE, Lyon, France; ^6^Armauer Hansen Research Institute, Addis Ababa, Ethiopia; ^7^UCD School of Veterinary Medicine and UCD Conway Institute, University College Dublin, Dublin, Ireland

**Keywords:** cattle, *Mycobacterium bovis*, *ex vivo*, precision cut lung slices, alveolar macrophages, type I interferon

## Abstract

Tuberculosis exacts a terrible toll on human and animal health. While *Mycobacterium tuberculosis* (Mtb) is restricted to humans, *Mycobacterium bovis* (Mb) is present in a large range of mammalian hosts. In cattle, bovine TB (bTB) is a noticeable disease responsible for important economic losses in developed countries and underestimated zoonosis in the developing world. Early interactions that take place between mycobacteria and the lung tissue early after aerosol infection govern the outcome of the disease. In cattle, these early steps remain poorly characterized. The precision-cut lung slice (PCLS) model preserves the structure and cell diversity of the lung. We developed this model in cattle in order to study the early lung response to mycobacterial infection. *In situ* imaging of PCLS infected with fluorescent Mb revealed bacilli in the alveolar compartment, in adjacent or inside alveolar macrophages, and in close contact with pneumocytes. We analyzed the global transcriptional lung inflammation signature following infection of PCLS with Mb and Mtb in two French beef breeds: Blonde d'Aquitaine and Charolaise. Whereas, lungs from the Blonde d'Aquitaine produced high levels of mediators of neutrophil and monocyte recruitment in response to infection, such signatures were not observed in the Charolaise in our study. In the Blonde d'Aquitaine lung, whereas the inflammatory response was highly induced by two Mb strains, AF2122 isolated from cattle in the UK and Mb3601 circulating in France, the response against two Mtb strains, H37Rv, the reference laboratory strain, and BTB1558, isolated from zebu in Ethiopia, was very low. Strikingly, the type I interferon pathway was only induced by Mb but not Mtb strains, indicating that this pathway may be involved in mycobacterial virulence and host tropism. Hence, the PCLS model in cattle is a valuable tool to deepen our understanding of early interactions between lung host cells and mycobacteria. It revealed striking differences between cattle breeds and mycobacterial strains. This model could help in deciphering biomarkers of resistance vs. susceptibility to bTB in cattle as such information is still critically needed for bovine genetic selection programs and would greatly help the global effort to eradicate bTB.

## Introduction

Bovine tuberculosis (bTB) caused by *Mycobacterium bovis* (Mb) remains one of the most challenging infections to control in cattle. Because of its zoonotic nature, this pathogen and its associated noticeable disease in cattle are under strict surveillance and regulation in the European Union. When bTB cases are detected through surveillance, culling of these reactor cattle is mandatory. In spite of intensive eradication campaigns, bTB is still prevalent in European cattle (1, 2) and has significant economic, social, and environmental implications. Since 2001, France is an officially bTB-free country, a status that was achieved through costly surveillance programs. However, each year, around 100 Mb foci of infection are identified (3), with certain geographical areas showing a constant rise in disease prevalence since 2004.

bTB eradication is an unmet priority that faces two major difficulties: the persistence of undetected infected animals in herds because of the lack of diagnostic sensitivity and the risk of transmission from infected sources (4). Moreover, the poor understanding of bTB pathophysiology in cattle and the lack of correlates of protection are substantial knowledge gaps that must be resolved so as to better tackle the disease (DISCONTOOLS, https://www.discontools.eu/).

Both Mb and *Mycobacterium tuberculosis* (Mtb) belong to the same genetic complex. Mtb is responsible for tuberculosis (TB) in humans, which displays similar features with bTB. It is estimated that one-third of the global human population are latently infected with Mtb, which kills 1.4 million people each year (5). Despite the high degree of identity that Mtb and Mb share both at the genetic level as well as during the infection process, the two pathogens display distinct tropism and virulence depending on the host. While Mb is highly virulent and pathogenic for cattle and a range of other mammals, Mtb is restricted to sustain in humans. An experimental infection of cattle with the widely used Mtb laboratory strain H37Rv, which was genome-sequenced in 1998 (6), shows a strong attenuation compared to Mb (7, 8). However, the natural infection of cattle with Mtb has been reported, and the strain Mtb BTB1558 was once such a case, isolated from a zebu bull in Ethiopia (9, 10). In comparison to the original UK Mb strain AF2122/97, the first genome-sequenced Mb isolate (11, 12), the Mtb strain BTB1558 displayed a much lower virulence in European cattle (13).

The Mb strains that circulate in France today are phylogenetically distant from the UK Mb reference strain. While AF2122 belongs to the European 1 clonal complex (14), the European 3 clonal complex is widespread in France, (15). The Eu3 genetic cluster is composed of field strains that share the SB0120 spoligotype with the attenuated Bacillus-Calmette-Guerin (BCG) vaccine strain (16, 17). In our study, we used Mb3601 as the representative strain of this widespread French cluster. Originally, Mb3601 was isolated from the tracheobronchial lymph node of an infected bovine in a bTB highly enzoonotic area in France (16). However, despite the widespread circulation in its original area, nothing is known today of the pathophysiology of Mb3601 infection.

Indeed greater knowledge is available on Mtb infection process and disease development both in humans and mouse models compared to Mb infection in cattle. With both mycobacteria, the alveolar macrophage (AMP) is the frontline cell that first presents the first niche for mycobacteria entering the lung, and the role of the AMP in early-stage infection is well established (8). Both Mtb and Mb have established their lifestyle in AMPs: they can escape its bactericidal mechanisms and multiply within this niche. During the infection process, bacilli disseminate to different anatomical sites and establish new infection foci both in the lungs and secondary lymphoid organs (18, 19). During Mtb infection, lung epithelial cells also play key roles in host defense [reviewed in (20–22)]. Type II pneumocytes are infected by Mtb (23) and produce pro-inflammatory cytokines which augment the AMP innate resistance mechanisms (24). The role of type II pneumocytes during Mb infection in cattle is not well known. Most of the available knowledge on the role of bovine macrophages (MPs) during Mb infection also comes from studies conducted with monocytes sampled from blood and derived as MPs during *in vitro* culture (25, 26).

In our study, we wanted to investigate the bovine innate response following Mb or Mtb infection in a preserved lung environment to allow the resident lung cells to interact with bacilli and crosstalk. Precision cut lung slices (PCLS) are an experimental model in which resident lung cell types are preserved and remain alive for at least 1 week (27). The tissue architecture and the interactions between the different cells are maintained. PCLS have already been validated for the study of various respiratory pathogens (27–29). In chicken PCLS, mononuclear cells are highly motile and actively phagocytic (30). This model is well designed to study complex interactions taking place early after the host–pathogen encounter. During Mb infection in cattle, important differences in the production of key proinflammatory cytokines such as IFNγ or TNFα by peripheral blood mononuclear cells are observed, depending on the clinical status of the animal. Interestingly, such differences are observed at early time points (31), indicating that the innate phase of the host response is key to the establishment of the pathological outcome of the infection.

Therefore, the PCLS model is ideally suited to investigate early host–pathogen interactions in the bovine lung during Mb infection and may help to find clues to the impact of the innate response on the outcome of infection. This model, which fully mimics the early environment of the bacillus entering the lung (compared to monocyte-derived MPs), may also aid in understanding the molecular basis of mycobacterial host preference (32). To this end, we decided to compare four mycobacterial strains: two Mtb species—namely, the Mtb H37Rv reference strain for human TB and the cattle derived Mtb BTB1558—and two Mb species—namely, Mb AF2122 as representative of the EU1 clonal complex and Mb3601 as the hallmark EU3 strain. Since the host genetic background also has a profound impact on the outcome of bTB disease (33), we decided to compare PCLS from two prevalent beef breeds in France—Charolaise and Blonde d'Aquitaine—and conducted a thorough characterization of the lung responses to Mb and Mtb during *ex vivo* infection. The PCLS allowed us to decipher important differences in the transcriptomic and cytokine profile during the innate response to infection, depending both on the breed, i.e., between Blonde d'Aquitaine and Charolaise cows, and on the mycobacterial species, i.e., between Mtb and Mb.

## Materials and Methods

### Animal Tissue Sampling

Lungs from 15 Blonde d'Aquitaine and nine Charolaise cows were collected post-mortem at a commercial abattoir. The animals were between 3 and 11 years old and originated from eight different French departments where no recent bTB outbreak had been noticed (Supplementary Figure 1). No ethical committee approval was necessary as no animal underwent any experimental procedure. After slaughter by professionals following the regulatory guidelines from the abattoir, the lungs from each cow were systematically inspected by veterinary services at the abattoir. The origin of each animal was controlled, and its sanitary status was recorded on its individual passport: the animals were certified to be free of bTB, leucosis, brucellosis, and infectious bovine rhinotracheitis.

### Bacterial Strains and Growth Conditions

Strains Mb AF2122/97 and Mb MB3601 had previously been isolated from infected cows in Great Britain and France, respectively (12, 15). The Mb3601-EGFP fluorescent strain was derived by electroporation with an integrative plasmid expressing EGFP and selected with Hygromycin B (50 μg/ml) (Sigma, USA) as described previously (34). Mtb BTB1558 had been previously isolated from a zebu bull in Ethiopia (13). Bacteria were grown in Middlebrook 7H9 broth (Difco, UK) supplemented with 10% BBLTM Middlebrook albumin–dextrose–catalase (BD, USA) and 0.05% Tween 80 (Sigma-Aldrich, St Louis, USA). At mid-log phase, the bacteria were harvested, aliquoted, and stored at −80°C. Batch titers were determined by plating serial dilutions on Middlebrook 7H11 agar supplemented with 10% oleic acid–albumin–dextrose–catalase (BD, USA), with 0.5% glycerol or 4.16 g/L sodium pyruvate (Sigma, USA) added for Mtb or Mb strains, respectively. The plates were incubated at 37°C for 3–4 weeks (H37Rv, BTB558, and AF2122) and up to 6 weeks for Mb3601 before colony-forming unit (CFU) numeration. The inocula were prepared from one frozen aliquot (titer determined by CFU numeration) that was thawed in 7H9 medium without glycerol and incubated overnight at 37°C. After centrifugation for 10 min at 3,000 × *g*, the concentration was adjusted to 10^6^ CFU/ml in RPMI medium.

### Obtention and Infection of Precision-Cut Lung Slices

PCLS were obtained from fresh lungs using a tissue slicer, MD 6000 (Alabama Research and Development). For each animal, the right accessory lobe was filled *via* the bronchus with RPMI containing 1.5% low-melting-point (LMP) agarose (Invitrogen) warmed at 39°C. After 20 min at 4°C, the solidified lung tissue was cut in 1.5-cm slices with a scalpel. A 0.8-mm diameter-punch was used to obtain biopsies that were placed in the microtome device of the Krumdieck apparatus, filled with cold phosphate-buffered saline (PBS), and 100-μm-thick PCLS were cut. One PCLS was introduced in each well of a 24-well plate (Nunc); 1 ml of RPMI 1640 (Gibco) supplemented with 10% heat-inactivated fetal calf serum (FCS, Gibco), 2 mM L-glutamine (Gibco), and PANTA™ antibiotic mixture (polymyxin B, amphotericin B, nalidixic acid, trimethoprim, and azlocillin; Becton Dickinson) was added to the well, and the plate was incubated at 37°C with 5% CO_2_. The medium was changed every 30 min during the first 2 h to remove all traces of LMP agarose. At 24 h later, after the last medium change, ciliary activity was observed under a microscope to ensure tissue viability.

The PCLS were infected for 2 days with 10^5^ CFU of Mb or Mtb strains. As indicated, the PCLS were either fixed in formalin for imaging or lysed with a Precellys in lysing matrix D tubes in 800 μl Tri-reagent for RNA extraction. The bacillary load of each strain present in the PCLS was compared after the transfer of the PCLS to a new plate at 1 day after infection (dpi), two washes in 1 ml of PBS, and homogenization in 1 ml of PBS in lysing matrix D tubes (MP Biomedicals) with a Precellys (Ozyme). To determine CFUs, serial dilutions were plated as described above.

### Alveolar Macrophages

To harvest alveolar macrophages (AMPs) from Blonde d'Aquitaine cows, broncho-alveolar lavages (BAL) were performed on the left basilar lobe of the lung at a local abattoir after culling the animal. The lobe was filled with 2 × 500 ml of cold PBS containing 2 mM EDTA (Sigma-Aldrich). After the massage, the BAL was collected and transported at 4°C to the laboratory. BAL was filtered with a 100-μm cell strainer (Falcon) and centrifuged for 10 min at 300 × *g*. The cells were washed in RPMI medium supplemented with 10% heat-inactivated fetal calf serum (Gibco), 2 mM L-glutamine (Gibco), and PANTA™ Antibiotic Mixture. Then, 10^7^ BAL cells per milliliter were suspended in 90% FCS and 10% dimethyl sulfoxide (Sigma-Aldrich) and cryopreserved in liquid nitrogen. At 1 day before infection, the BAL cells were thawed at 37°C, washed in complete RPMI medium, and transferred to a 75-cm^2^ culture flask with a ventilated cap. After 2 h at 37°C and 5% CO_2_, non-adherent cells were removed, and adherent AMPs were incubated 2 × 10 min at 4°C with 10 ml of cold PBS to detach and enumerate them in a Malassez chamber. Then, 5 × 10^5^ AMPs/well were distributed in a 24-well plate and incubated overnight at 37°C and 5% CO_2_. The medium was changed once, and AMPs were infected with Mb3601 or Mtb H37Rv at a multiplicity of infection (MOI) of 1. At 6 and 24 h post-infection, the supernatants were filtered through a 0.2-μm filter, and the cells were lysed in 800 μl of Tri-reagent for RNA extraction. The MOI was checked by CFU determination at 24 h after infection.

### Cell Supernatant Collection and Lactate Dehydrogenase Assay

In order to evaluate cytotoxicity, supernatants from infected PCLS or AMPs were passed through a 0.2-μm filter at indicated time points, and cells were lysed in 1 ml of lysis buffer (5 mM EDTA, 150 mM NaCl, 50 mM Tris-HCl, Triton 1%, pH 7.4), containing anti-proteases (Roche), in a lysing matrix D tube, with a Precellys apparatus. The homogenates were clarified by centrifugation for 10 min at 10,000 × *g*, filtered through 0.2 μm, and collected on microplates. The cytotoxicity of infection in PCLS was assessed using the Non-radioactive Cytotoxicity Assay kit (Promega) according to the manufacturer's instructions. The cytotoxicity was calculated as cytotoxicity (%) = [OD_490_ of lactate dehydrogenase (LDH) in the supernatant]/(OD_490_ of LDH in the supernatant + OD_490_ of LDH in the PCLS homogenates) × 100.

### Immunohistochemistry on PCLS

The infected PCLS were fixed 24 h at 4°C with 4% formalin and then transferred to a 48-well culture plate in PBS. All steps that will be described below were done under gentle agitation at room temperature (RT). The PCLS were incubated for 2 h with 100 μl of PBS, 0.25% Triton X-100, and 10% horse serum for permeabilization and saturation (saturation buffer). They were incubated overnight at 4°C with primary Ab (anti-bovine MHCII clone MCA5655 from BioRad and anti-bovine pancytokeratine clone BM4068 from Acris) diluted in saturation buffer. The PCLS were washed four times with 300 μl of PBS (two times for 5 min and then two times for 10 min) and then incubated for 3 h with fluorescent-conjugated secondary antibodies diluted in saturation buffer (goat anti-mouse IgG1-APC and goat anti-mouse IgG2a A555 from Invitrogen). The PCLS were washed four times with 300 μl of PBS (two times for 5 min and then two times for 10 min), transferred on cover slides which were mounted with Fluoromount-G™ mounting medium containing DAPI (Invitrogen), and sealed with a transparent nail polish. Z-stack imaging was performed at ×63 enlargement with a confocal microscope (LEICA) and analyzed with LAS software. The presence/absence of Mb and number of macrophages per alveoli were numerated by eye at the confocal microscope, with one person counting and the other confirming and reporting the data.

### Quantification of Cytokines and Chemokines Released by PCLS and AMPs

The cytokine and chemokine levels produced by PCLS after 2 dpi were assessed in a Multiplex assay in supernatants (dilution 1:2) with MILLIPLEX® Bovine cytokine/chemokine panel 1 (BCYT1-33K-PX15, Merck) according to the manufacturer's instructions. IFNγ, IL-1α, IL-1β, IL-4, IL-6, IL-8 (CXCL8), IL-10, IL-17A, IL-36RA (IL-1F5), IP-10 (CXCL10), MCP-1 (CCL2), MIP-1α (CCL3), MIP-1β (CCL4), TNFα, and VEGF-A were measured. Data were acquired using a MagPix instrument (Luminex) and analyzed with Bio-Plex Manager software (Bio-Rad). IL-8 was out of range in the Multiplex, so we performed a sandwich ELISA with the following references: goat anti-bovine interleukin-8 Ab AHP2817, recombinant bovine interleukin-8 PBP039, and goat anti-bovine interleukin-8 Ab conjugated to biotin AHP2817B (all from Bio-Rad), following the protocol according to the manufacturer's instructions.

### RNA Extraction and Gene Expression Analysis

The total RNA from two pooled PCLS was extracted using a MagMAX™-96 Total RNA isolation kit (ThermoFisher). For AMPs, we used the Nucleospin RNA isolation kit (Macherey Nagel). After DNase treatment (ThermoFisher or Macherey Nagel), the mRNAs were reverse-transcribed with iScript™ Reverse Transcriptase mix (Biorad) according to the manufacturer's instructions. The primers (Eurogenetec; Supplementary Table 1) were validated, using a serially diluted pool of cDNA mix obtained from bovine lung, lymph nodes, blood, and bone marrow, with a LightCycler® 480 Real-Time PCR System (Roche). Gene expression was then assessed with the BioMark HD (Fluidigm) in 96 × 96-well integrated fluidic circuit plate according to the manufacturer's instructions. The annealing temperature was 60°C. The data were analyzed with Fluidigm RealTime PCR software to determine the cycle threshold (Ct) values. The messenger RNA (mRNA) expression was normalized to the mean expression of three housekeeping genes (*PPIA, GAPDH*, and *ACTB*) to obtain the ΔCt value. For each animal, values from infected PCLS were normalized to the uninfected PCLS gene expression (ΔΔCt value and relative quantity = 2 ^−Δ*ΔCt*^). Principal component analysis (PCA) was performed using ΔΔCt values in R studio (version 1.1.456, ©2009–2018 RStudio, PBC) using the FactoMineR packages (version R 3.5.3).

### Statistical Analysis

The individual data and the median and interquartile range are presented in the figures, except for **Figure 2** where the mean and standard error of the mean (SEM) are presented. Statistical analyses were performed with Prism 6.0 software (GraphPad). Analyses were performed on data from two to six independent experiments, with two-way ANOVA or Wilcoxon non-parametric tests for paired samples used. The represented *p*-values were *p < 0.05, **p < 0.01, and ***p < 0.001.

## Results

### *Ex vivo* Infection With Mycobacteria of Live Bovine Lung Tissue in PCLS Allows Bacilli Uptake by AMPs and Their Recruitment to the Alveoli

The early events of bTB pathophysiology in the bovine lung remain poorly defined due to the complexity of biocontained experimental infection in large animals. Since PCLS have been used to study viral respiratory infections in the bovine (27), we decided to use this model to assess early events taking place following entry of Mb into the lung. We infected bovine PCLS obtained *ex vivo* with the four mycobacterial strains: Mb AF2122, Mb3601, Mtb H37Rv, or BTB1558.

**Figure 1:**
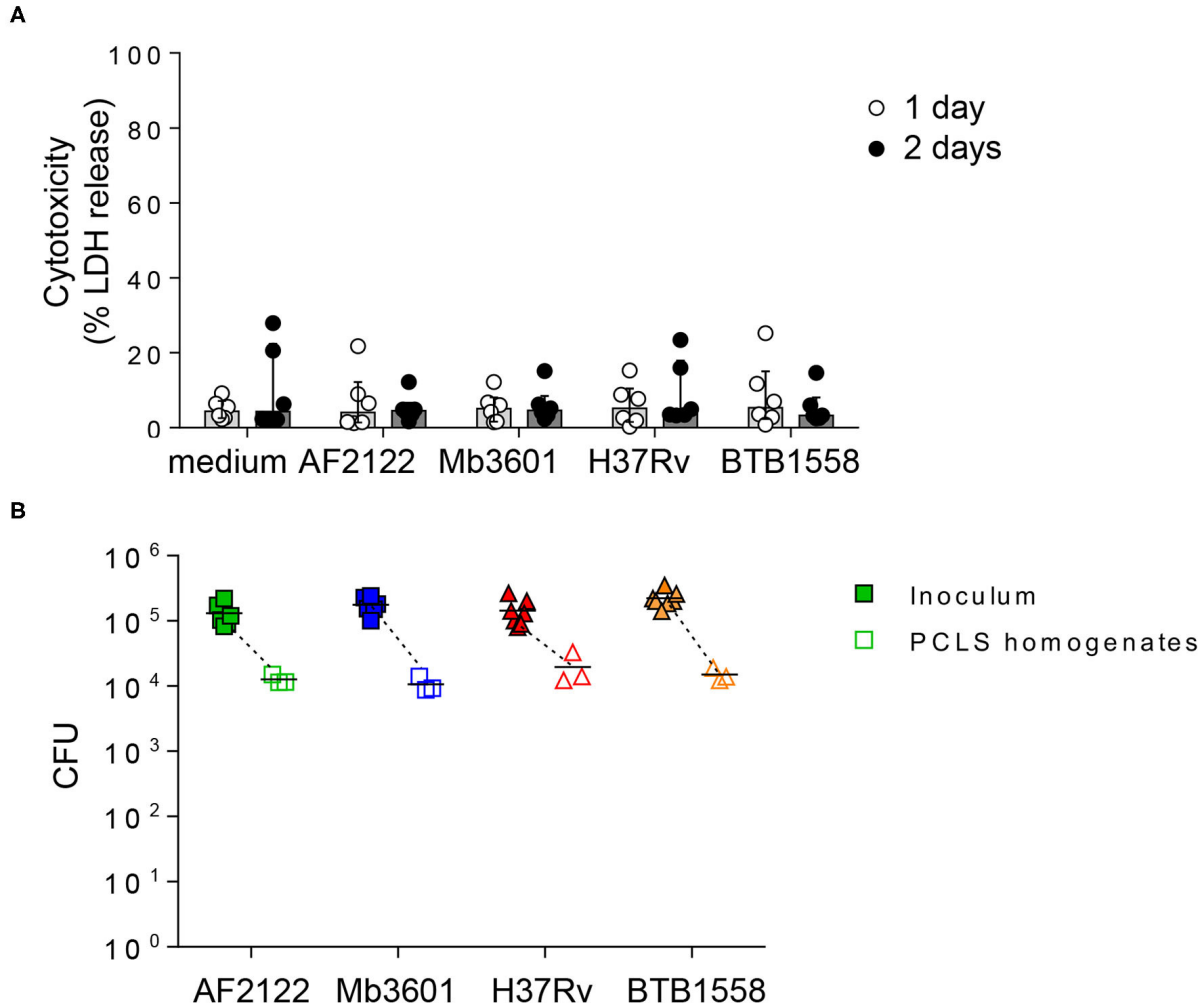
Precision-cut lung slice (PCLS) infection with four different Mb or Mtb strains does not induce lung tissue cytotoxicity, and equivalent numbers of bacilli are recovered 24 h post-infection. **(A)** PCLS prepared from Blonde d'Aquitaine lungs post-mortem were infected with 10^5^ CFU of two Mb strains (AF2122 or Mb3601) or two Mtb strains (H37Rv or BTB1558). After 1 and 2 days post-infection, the PCLS supernatants were harvested, and tissue was homogenized. Lactate dehydrogenase (LDH) was measured in both compartments using the “non-radioactive cytotoxicity assay” kit. Cytotoxicity was determined as (%) = (O.D. 490 nm LDH in supernatant)/(O.D. 490 nm LDH in supernatant + O.D. 490 nm LDH in PCLS homogenates) × 100. Individual data and the median and interquartile range in each group are presented (*n* = 6 animals from six independent experiments). **(B)** At 24 h post-infection, the PCLS were washed and homogenized to recover bacilli. The inoculum and PCLS homogenates were serially diluted and plated with colony-forming units numerated after 3–6 weeks of incubation. Individual data and the mean in each group are presented (*n* = 6 independent inocula prepared; PCLS homogenates data represent the mean of technical duplicates from *n* = 3 animals from three independent experiments).

We first monitored tissue cytotoxicity at 1 and 2 dpi using a LDH release assay. The mean percentage of cytotoxicity remained below 10%, and no difference was observed between infected and non-infected PCLS (Figure 1A). The ciliary activity from the PCLS bronchial cells monitored every day under a light microscope remained vigorous and stable after infection (data not shown). We calibrated our model and inocula to use 10^5^ CFUs for each of the four different strains. We analyzed CFUs still present in PCLS at 24 h later and observed an equivalent 1 log decrease for all strains (Figure 1B). This indicated an equivalent infection by all strains, allowing them to be directly compared. Therefore, with a similar bacterial load and excellent tissue viability in all experimental conditions, we validated PCLS as a model to study the early events taking place in the bovine lung after infection with mycobacteria.

**Figure 2:**
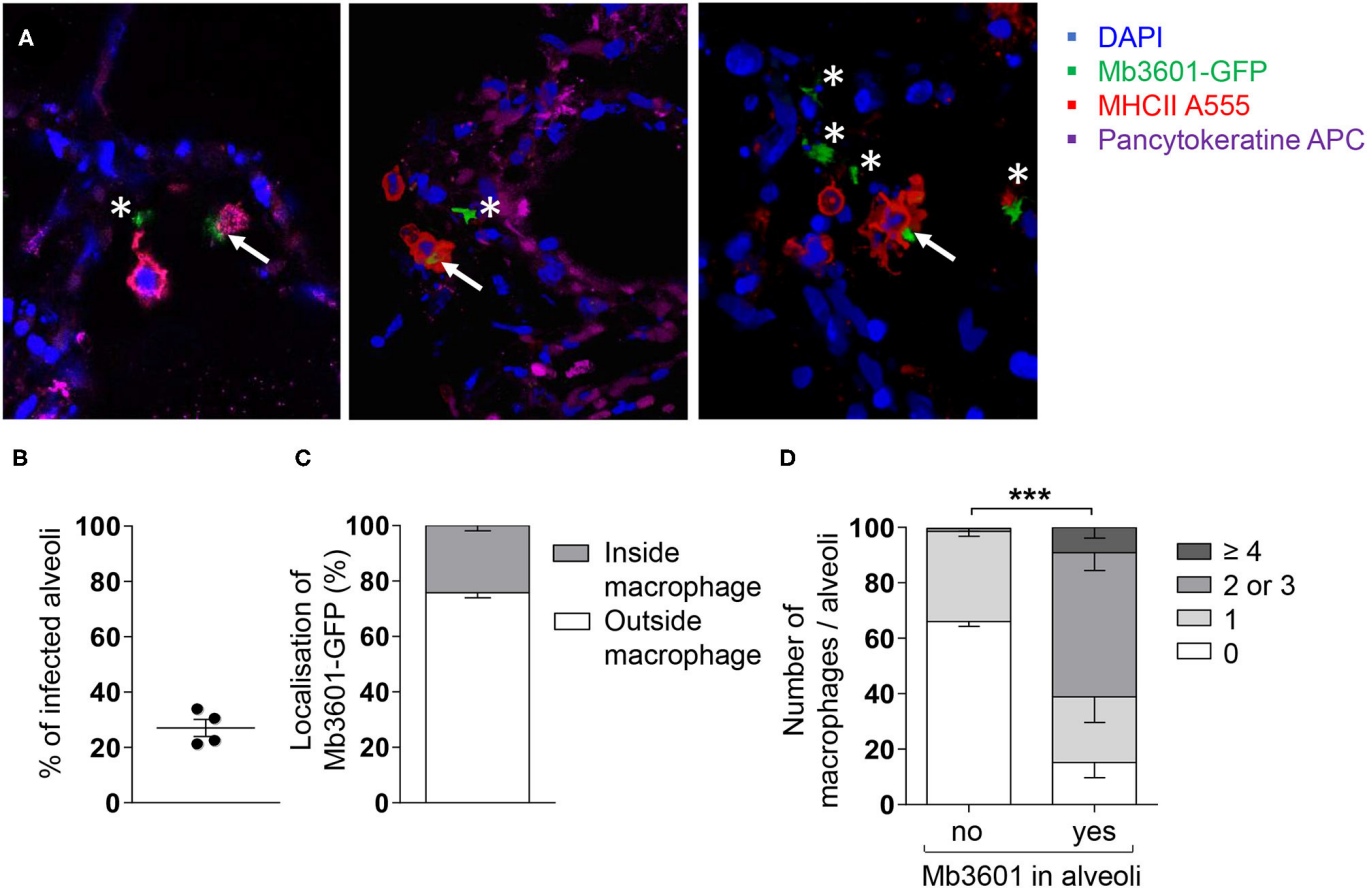
Mb3601 is internalized by alveolar macrophages (AMPs) in the preserved lung structure from precision-cut lung slice (PCLS), and the infected alveoli contain higher numbers of AMPs compared to non-infected alveoli. The PCLS were infected with 10^5^ colony-forming units of the green fluorescent protein Mb3601-GFP recombinant strain and fixed 2 days later. After labeling with anti-pancytokeratine (magenta) and anti-MHCII antibodies (Alexa 555, red), the PCLS were mounted with Fluoromount-G™ mounting medium containing DAPI (blue) and analyzed under a Leica confocal microscope **(A)**; 3D images were analyzed with Leica LAS software. Z-stack imaging was performed at × 63 enlargement (10–15 μm in thickness, step size of 0.5–1 μm). The white asterisks indicate extracellular bacilli, and the white arrows indicate bacilli inside MHC-II^pos^ AMPs. **(B)** The graph represents the percentage of infected alveoli per PCLS among the 55–80 alveoli that were observed under the microscope (*n* = 4 PCLS from two different Blonde d'Aquitaine cattle). **(C)** Stack histogram of the mean percentage ± SEM of intra- or extracellular bacilli among a minimum of 15 infected alveoli that were observed (*N* = 4 PCLS). **(D)** The number of MHC-II^pos^ AMPs per alveoli was counted in infected or non-infected alveoli. The data presented as percent are the mean ± SEM of *n* = 4 PCLS from two different Blonde d'Aquitaine cattle. Between 55 and 80 alveoli were observed to obtain these data (two-way ANOVA, ***p < 0.001).

In order to visualize the interactions taking place between bacilli and lung cells, we infected the PCLS with a fluorescent version of the Mb3601 strain, and at 1 and 2 dpi, we analyzed the cells by *in situ* immunohistochemistry. The lung structure was visualized by DAPI and pancytokeratine staining, and we used confocal microscopy to image 10–15-μm sections and localize Mb3601-EGFP (Figure 2A). We observed Mb in 27 ± 3% of PCLS alveoli (Figures 2A,B) and almost always in close contact with large MHC-II-positive AMPs. The bacilli were localized outside AMPs in 76 ± 2% observations and resided intracellularly in AMPs in 24 ± 2% (Figures 2A,C and Supplementary Video 1). Interestingly, the number of AMPs per alveoli differed upon bacilli presence or absence (Figure 2D). In uninfected PCLS, lung alveoli generally contained one AMP (data not shown). However, in Mb-infected PCLS, we either observed no AMPs in 66 ± 2% of alveoli or one AMP in 33 ± 2% of alveoli in the absence of any Mb. On the contrary, the number of AMPs significantly increased in alveoli where at least one Mb was observed (Figure 2D, *p* < 0.001). The number of AMPs varied among infected alveoli, with 24 ± 9% containing one AMP, 52 ± 6% containing two or three AMPs, and 9 ± 4% containing more than four AMPs. Such observations indicated that, during the 2 days of infection, AMPs were recruited from one alveolus to another in response to signals linked to Mb infection. In conclusion, even though Mb infection was performed *ex vivo*, bacilli were observed in the alveoli, close or inside their target host cell, i.e., the AMP. Moreover, the PCLS model was physiological enough to allow AMPs to crawl in response to signals linked to bacilli entry.

### The Lung Response to Mycobacterial Infection Vastly Differs Between Blonde d'Aquitaine and Charolaise Cows

**Figure 3:**
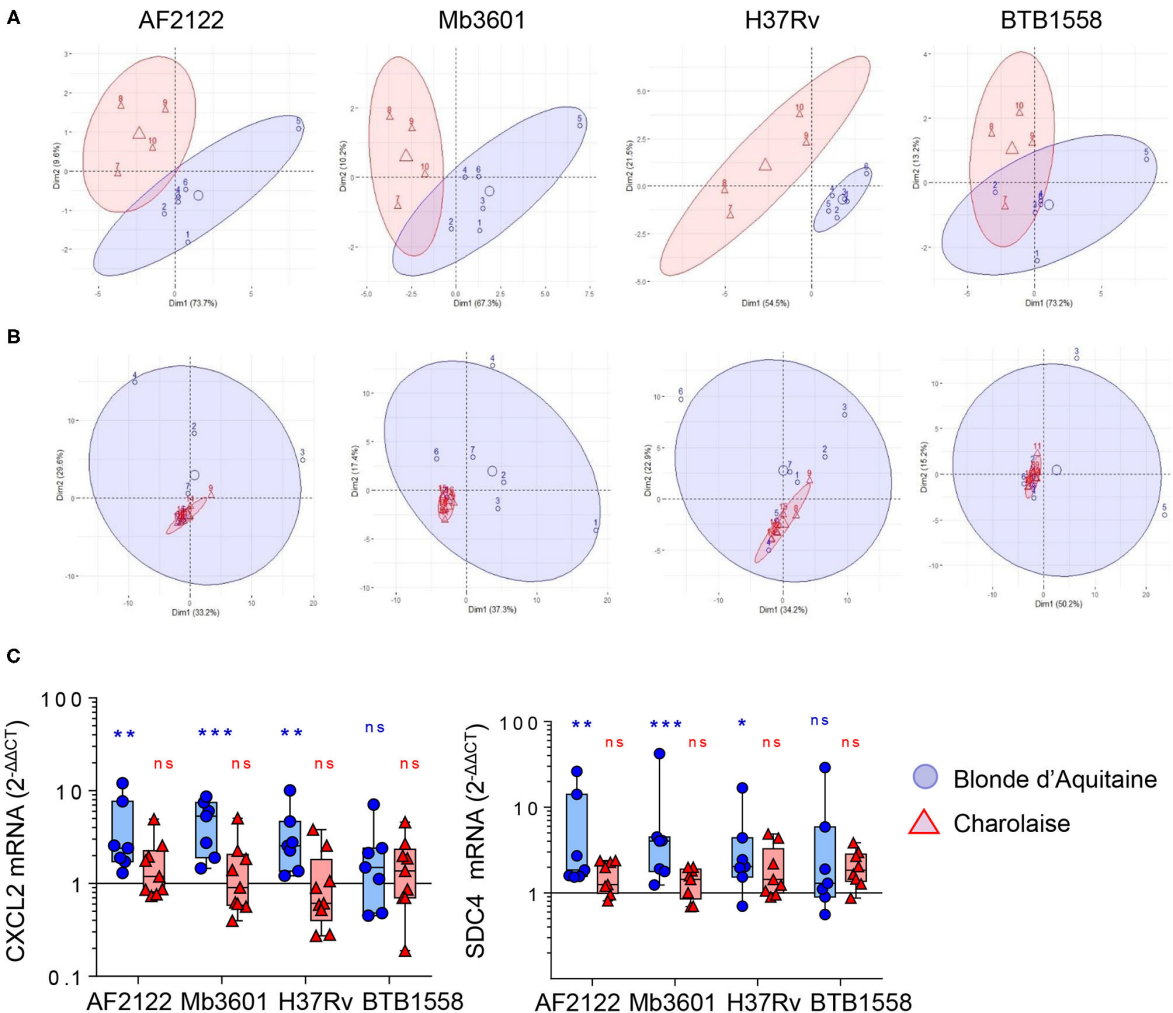
Principal component analysis (PCA) of inflammatory lung tissue signature reveals differences between two beef cattle breeds after 2 days of infection by Mb or Mtb. **(A)** Fifteen cytokines and chemokines were measured in PCLS supernatants from Blonde d'Aquitaine or Charolaise cows 2 days after infection with four different mycobacterial strains. Raw data were used to run PCA in R studio. Individual data are shown (*n* = 4 for Charolaise, red; *n* = 6 for Blonde d'Aquitaine, blue). The ellipses represent a confidence range of 90%. **(B)** PCA were built from the expression data of 96 genes (2^−Δ*ΔCt*^) obtained from precision-cut lung slice total RNA extracted 2 days after infection. Individual data are shown (*n* = 9 for Charolaise, red; *n* = 7 for Blonde d'Aquitaine, blue). The ellipses represent a confidence range of 90%. **(C)** Two examples of differentially expressed genes. Individual data and the median and interquartile range in each group are presented (*n* = 7 Blonde d'Aquitaine and *n* = 9 Charolaise) *p < 0.05; **p < 0.01; ***p < 0.001. Two-way ANOVA test.

Two bovine beef breeds are widely used in France: Blonde d'Aquitaine and Charolaise. We decided to compare how these two breeds respond to mycobacterial infection, using our PCLS system. We measured 15 cytokines and chemokines secreted by the lung tissue at 2 dpi with the four mycobacterial strains and performed a PCA. As depicted in Figure 3A, the PCA revealed important differences in the immune response of the lung tissue between the two breeds. The group samples clearly plotted apart, and their ellipses showed either a small overlay (AF2122 and Mb360A) or no overlay at all (H37Rv). The results for the BTB1558 group showed less clustering of samples due to higher individual variations. We then extracted total RNA from PCLS after 1 or 2 dpi and analyzed the expression of 96 genes related to innate immunity and inflammation (see the full list in Supplementary Table 1). The RT-qPCR data were normalized and expressed as fold change compared to uninfected PCLS control for each cow. Gene expression was higher at 2 days after infection compared to that at 1 dpi (data not shown). We therefore decided to focus our analysis on this 2-dpi time point. Remarkably, the transcriptomic signature induced by infection was very low for the Charolaise breed, whichever mycobacterial strain was used, which explains the clustering of Charolaise samples (Figure 3B). Increasing the inoculum in the Charolaise PCLS up to 5 × 10^6^ CFU did not induce gene expression (Supplementary Figure 2). The response of the lung tissue to mycobacterial infection in Blonde d'Aquitaine was very different compared to that in Charolaise as revealed by a PCA (Figure 3B). Whereas, in PCLS from Charolaise the gene expression from infected and non-infected controls clustered, in PCLS from Blonde d'Aquitaine, the gene expression levels were significantly more dispersed after infection compared to those of controls (Figure 3B). We compared the individual gene expression between the two breeds for a number of genes. For instance, both the *CXCL2* chemokine and the mycobacteria receptor syndecan 4 *SDC4* were significantly upregulated after PCLS infection with AF2122, Mb3601, or H37Rv in Blonde d'Aquitaine, but not in Charolaise (Figure 3C). Our data altogether revealed important differences in the early lung response to mycobacterial infection, depending on the breed of the animals, that could be measured both at the gene expression and protein production level in the PCLS system.

### The Overall Inflammation Signature in the Lung Tissue Is Triggered More Efficiently by *M. bovis* Than *M. tuberculosis*

**Figure 4:**
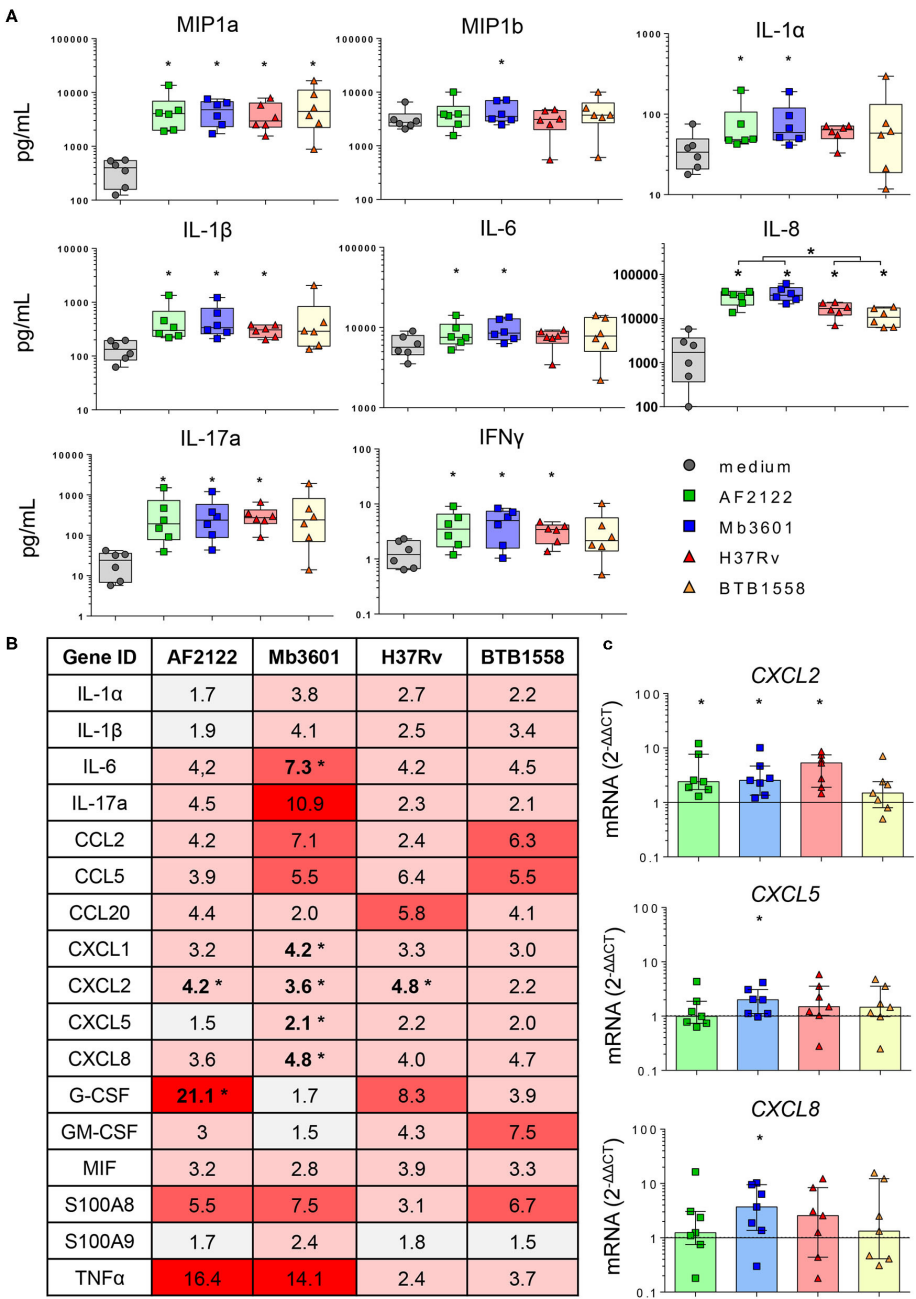
The lung inflammatory neutrophil and monocyte recruitment signature induced by infection in precision-cut lung slice (PCLS) from Blonde d'Aquitaine cows is more efficiently triggered by *Mycobacterium bovis* than *Mycobacterium tuberculosis*. **(A)** The cytokine and chemokine levels were measured in PCLS supernatant by Multiplex ELISA 2 days after infection with two Mb or two Mtb strains. Individual data and the median and interquartile range in each group are presented (*n* = 6 cows). **(B)** Table of the mean of fold change (2^−Δ*ΔCT*^) for each group (*n* = 7 cows) of 17 major genes involved in neutrophil and monocyte recruitment and inflammation. The graduated red box coloring represents levels of gene expression, and the asterisks mark significant differences compared to non-infected controls. **(C)** *CXCL2, CXCL5*, and *CXCL8* gene expression at 2 days post-infection. Individual data and the median and interquartile range in each group are presented (*n* = 7 cows). **(B,C)** *p < 0.05 (Wilcoxon nonparametric test).

We then focused our analysis on Blonde d'Aquitaine to determine how the lung tissue responded to different mycobacterial strains. We analyzed 15 cytokines and chemokines produced in the PCLS supernatants 2 days following an infection. No IL-4 was detected, and the production of TNFα, IL-36RA, IL-10, VEGFA or MCP-1 was not different between infected PCLS and controls (Supplementary Figure 3A). We observed that *ex vivo* infection of PCLS with mycobacteria triggered an inflammatory response that contrasted between the strains (Figure 4A). At the protein level, the Mtb strain BTB1558 induced the most heterogenous response, and due to high individual variation, differences in chemokine/cytokine production between infected PCLS and controls only reached a statistical significance for MIP-1a (CCL3) and IL-8 (Figure 4A). These two inflammatory mediators were also strongly induced by all strains. IL-17A, IL-1β, and IFNγ were efficiently induced by mycobacterial infection, and no significant difference was observed between Mtb and Mb. By contrast, IL-6 and IL-1α were significantly induced after Mb, but not Mtb, infection, and IL-8 production was also significantly higher after Mb than Mtb infection (Figure 4A). The only strain able to induce a significant production of MIP-1b was Mb3601. We then analyzed the inflammatory transcriptomic signature using a panel of 17 genes involved in monocyte/macrophage and neutrophil recruitment (Figure 4B). A number of these genes was significantly upregulated upon PCLS infection even though significant differences were not always reached due to inter-individual variation. Remarkably, Mb3601 induced the strongest inflammatory response, with five out of 17 genes significantly upregulated compared to non-infected controls. Focusing on chemokines involved in neutrophil recruitment, we observed that *CXCL2* expression was induced by all strains—except BTB1558—whereas *CXCL1, CXCL5*, and *CXCL8* were only upregulated by Mb3601 (Figures 4B,C). *IL-6* expression was also high after Mb3601 infection. Therefore, the *ex vivo* infection of PCLS efficiently triggered signals involved in monocyte and neutrophil recruitment. Infection by Mb strains, more specifically the Mb3601 strain circulating in France, triggered inflammation in the bovine lung more efficiently than Mtb.

### The Type I Interferon Pathway Is Induced in the Bovine Lung by Infection With *M. bovis*, but Not *M. tuberculosis*

**Figure 5:**
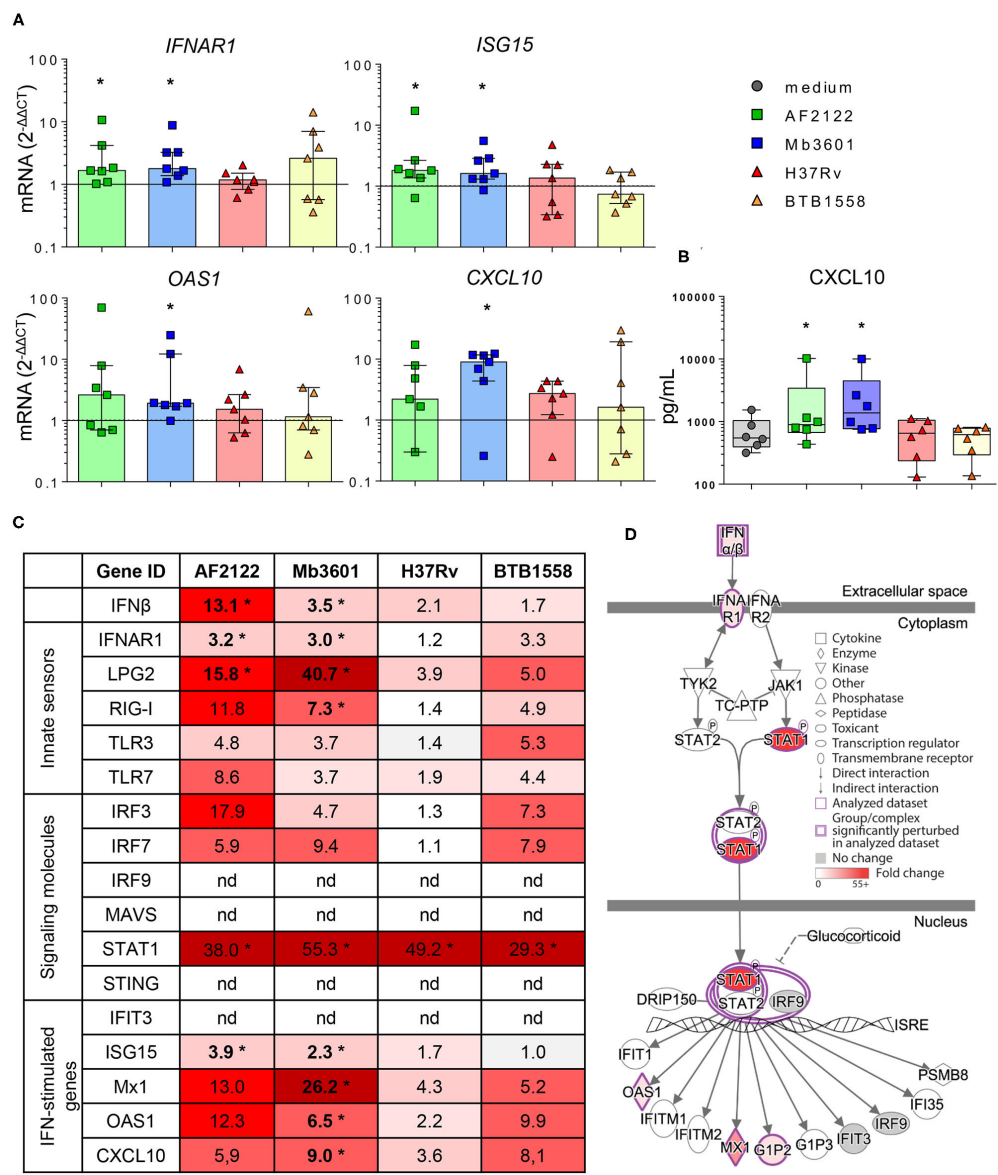
Mb but not Mtb infection in the lung tissue from Blonde d'Aquitaine cows induces the type I interferon pathway. The precision-cut lung slice (PCLS) was infected as described in Figure 1. **(A)** *IFNAR*, ISG15*, CXCL10*, and *OAS1* gene expression at 2 dpi. Individual data and the median and interquartile range in each group are presented (*n* = 7). **(B)** CXCL10 protein level was measured in PCLS supernatant at 2 dpi. Individual data and the median and interquartile range in each group are presented (*n* = 6). **(C)** The table represents the mean of fold change (2^−*dCT*^) for each group (*n* = 7) of major genes involved in type I interferon pathway. The graduated red box coloring is for higher gene expression, and the asterisks mark significant differences compared to uninfected PCLS. nd, not detected. **(D)** Ingenuity pathway analysis drawing of the type I interferon pathway under IFNAR in the Mb3601 group. The graduated red box coloring is for higher gene expression. **(A–C)** *p < 0.05 (Wilcoxon nonparametric test).

Because in humans and mouse models susceptibility to mycobacterial infection and disease progression is driven by type I IFN (35–37), we decided to compare the induction of this pathway by Mtb and Mb strains in bovine lung tissue. We measured the expression of different genes involved in the type I IFN pathway in Blonde d'Aquitaine PCLS infected by the four mycobacterial strains (Figure 5). The gene expression of both *IFN*β and the *IFNAR1* receptor was significantly increased after Mb but not Mtb infection (Figures 5A,C). Similarly, the major IFN-stimulated genes (ISG) *MX1, OAS1, ISG15*, and *CXCL10* were induced only after Mb infection (Figures 5A,C), and this difference was also detected at the protein level for CXCL10 (Figures 5A,B). Therefore, we observed the induction of a number of genes of the type I IFN pathway, recapitulated in Figure 5D, after infection with Mb, but not Mtb, strains. Strikingly, strain Mb3601 was the highest inducer of this pathway in the lung from Blonde d'Aquitaine cows.

**Figure 6:**
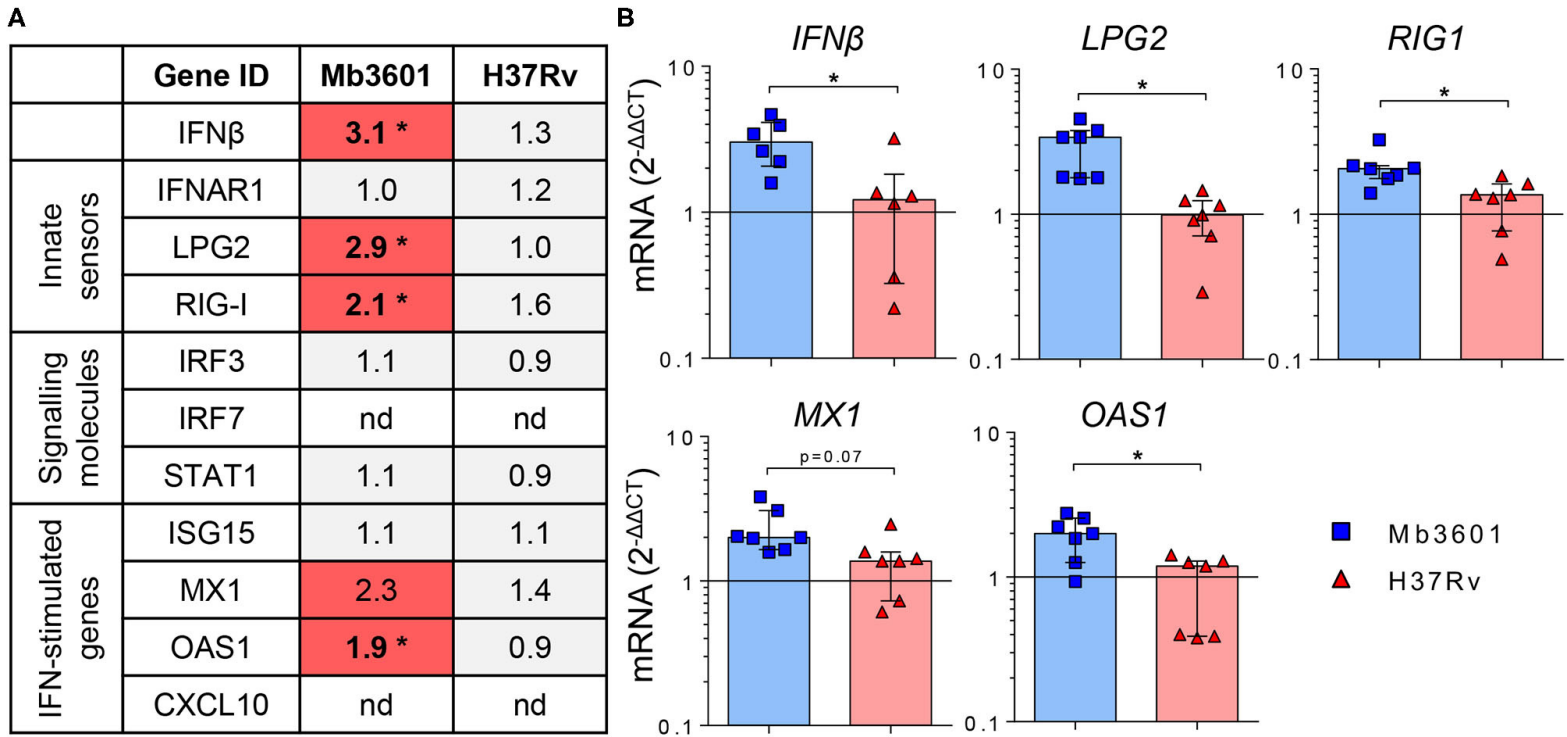
Alveolar macrophages (AMP) from Blonde d'Aquitaine contribute to the type I IFN signature in the lung induced by Mb infection. AMPs from Blonde d'Aquitaine lungs were infected with 10^5^ CFU of Mb3601 or Mtb H37Rv. At 6 h later, mRNA was extracted, and the expression of major genes from the type 1 IFN pathway was analyzed. **(A)** Mean fold change (2^−Δ*ΔCT*^) of gene expression normalized to three housekeeping genes was calculated in each group (*n* = 7). The graduated red box coloring represents gene expression, and the asterisks mark significant differences compared to non-infected controls (nd, not detected). **(B)** *IFN*β, *LPG2, RIG1, MX1*, and *OAS1* gene expression in AMPs was analyzed by RT-qPCR at 6 h post-infection. Individual data and the median and interquartile range in each group are presented (*n* = 7). **(A,B)** *p < 0.05 (Wilcoxon nonparametric test).

Because AMPs are the most prominent host cells interacting with Mb (8), which we also observed in PCLS (Figure 2), we next decided to decipher if AMPs contributed to the induction of the type I IFN pathway after Mb3601 or H37Rv infection. At 1 day after the infection of AMPs with these two strains, similar bacterial levels were recovered (data not shown). At 6 h post-infection, no cell cytotoxicity was observed, and we analyzed the expression of genes from the type 1 IFN pathway at this early time point. While we did not observe differences in *IFNAR1, IRF3, STAT1*, nor *ISG15* expression induced by the two strains (Figure 6A), *IFN*β*, LPG2, RIG1*, and *OAS1* were significantly induced after infection with Mb3601, but not H37Rv (Figure 6B). Regarding *MX1*, the same trend was observed, although statistical significance was not reached (Figure 6B, *p* = 0.07).

Interestingly, while CXCL10 was detected both at the mRNA and protein levels in PCLS infected with Mb (Figure 5), we did not detect the expression of this gene by AMPs in our analysis. These results altogether demonstrate that AMPs globally contribute to the type I IFN pathway in the lung after Mb infection, although other cells present in PCLS may also specifically induce some genes, such as *CXCL10* or *IRF7*, for example (Figures 5C, 6A).

## Discussion

The lung is the main organ targeted by Mb infection in cattle (38), and early interactions between the different lung cell types and the bacillus that govern the pathophysiology of the disease need to be better understood. In this study, we used PCLS for the first time to monitor the early bovine lung response to Mb infection and validated this model as a means to measure the local innate response at the protein and mRNA level. A main advantage of PCLS is conservation of the complex lung tissue both in structure and diversity of cell types. After infection with mycobacteria, the ciliary activity of bronchial cells was maintained. The AMP main function is to patrol the lung, crawling in and between alveoli; they sensed, chemotaxed, and phagocytosed debris or inhaled bacteria (39). We observed increased numbers of AMPs in alveoli where Mb was present, indicating AMP mobility inside the tissue. In chicken, PCLS allowed the observation of the movement of macrophages and phagocytosis (30). The AMP is well established as the main host cell for Mtb infection in humans (40) and Mb infection in cattle (41). Accordingly, in PCLS, we observed Mb inside AMPs in 20% of infected alveoli. We sometimes observed several bacilli inside one AMP. Although Mb is able to replicate inside this hostile cell, it is difficult to know if this observation was due to bacillary multiplication or the phagocytosis of several bacilli. This issue would need live imaging of PCLS to follow the fate of fluorescent Mb, an approach which remains challenging under BSL3 conditions.

In uninfected PCLS, we observed generally one AMP for two to three alveoli [Supplementary Figure 4; in good correlation with the observations of Neupane et al. (39)]. After Mb infection, we observed several AMPs inside the same alveolus in 50% of cases. Moreover, when the alveoli contained more than four AMPs, they were in close contact. Multinucleated giant cells are formed by the fusion of several MPs and are a hallmark of TB pathophysiology. It has recently been demonstrated that, after infection of human or bovine blood-derived MPs by Mb or Mtb, only Mb was able to induce the formation of multinucleated cells (26). Although at 2 dpi we did not observe the formation of such cells in PCLS, it would be interesting to analyze if such events could be detected after longer infection periods. Goris et al. have maintained bovine PCLS during 1 week to study viral infections (27).

One other advantage of our model is the preserved diversity of lung cell composition. PCLS contain type I and II pneumocytes, endothelial cells, and bronchial cells (Supplementary Figure 4) and also produce key molecules like surfactant, which has an established role in Mtb uptake (42). Mtb is also capable of invading type II alveolar epithelial cells (23) that play important roles in host defense (20–22). In our study, we did not observe intraepithelial Mb, but specific labeling of bovine epithelial cells would be required to investigate interactions between bovine lung pneumocytes and Mb in more detail. However, as we have observed that infected AMPs were in close contact with epithelial cells in PCLS, this model will allow a more refined analysis of the crosstalk between AMPs and pneumocytes during Mb infection (24).

One limitation of the PCLS model is the lack of recruitment of immune cells from circulating blood. During mycobacterial infection, in response to local signals, a variety of immune cells are recruited to the infection site to form the mature granuloma that constrains bacillary multiplication. How this response is orchestrated at the level of the lung tissue in cattle remains poorly established. Neutrophils, together with other innate cells, such as macrophages, γδ-T lymphocytes, and natural killer cells, were recently identified as key immune cells in the early containment of infection (43) and development of early lesions (44). Moreover, humans regularly exposed to Mtb or cattle exposed to Mb do not always develop signs of infection, i.e., remain negative in IFNg-release assay or skin testing. In humans, such resistance to infection through the successful elimination of bacilli could be mediated by neutrophils (45). Similarly, in cattle experimentally infected with Mb, some contact animals resist infection, while others develop lesions due to productive infection (46). It is possible that neutrophils could also play an important role in the early elimination of Mb in cattle (43). Immune signals involved in the early recruitment of neutrophils to the lung after the entry of Mb need to be better understood in cattle. It is known that epithelial cells secrete, among other cytokines and chemokines, MIP1 and CXCL8 that attract MPs and neutrophils to the site of infection. Interestingly, we measured important differences in the production of such mediators by PCLS in response to different strains of mycobacteria that could be linked to variable virulence. Although one cattle type II pneumocyte cell line has been described (47), such transformed cells are less physiologically relevant than primary cells. Recently, immortalized type II cells were co-cultured with endothelial cells as a model of the bovine alveolus to study mycobacterial interactions with BCG. In this study, the authors detected the production of IL-8, TNFα, IL-22, and IL-17a. One limitation of this model was epithelial cell death, which occurred shortly after infection (48). As a physiological model, PCLS could help in understanding the early orchestration of the local inflammatory response in the lung in response to mycobacterial infection.

**Figure 7:**
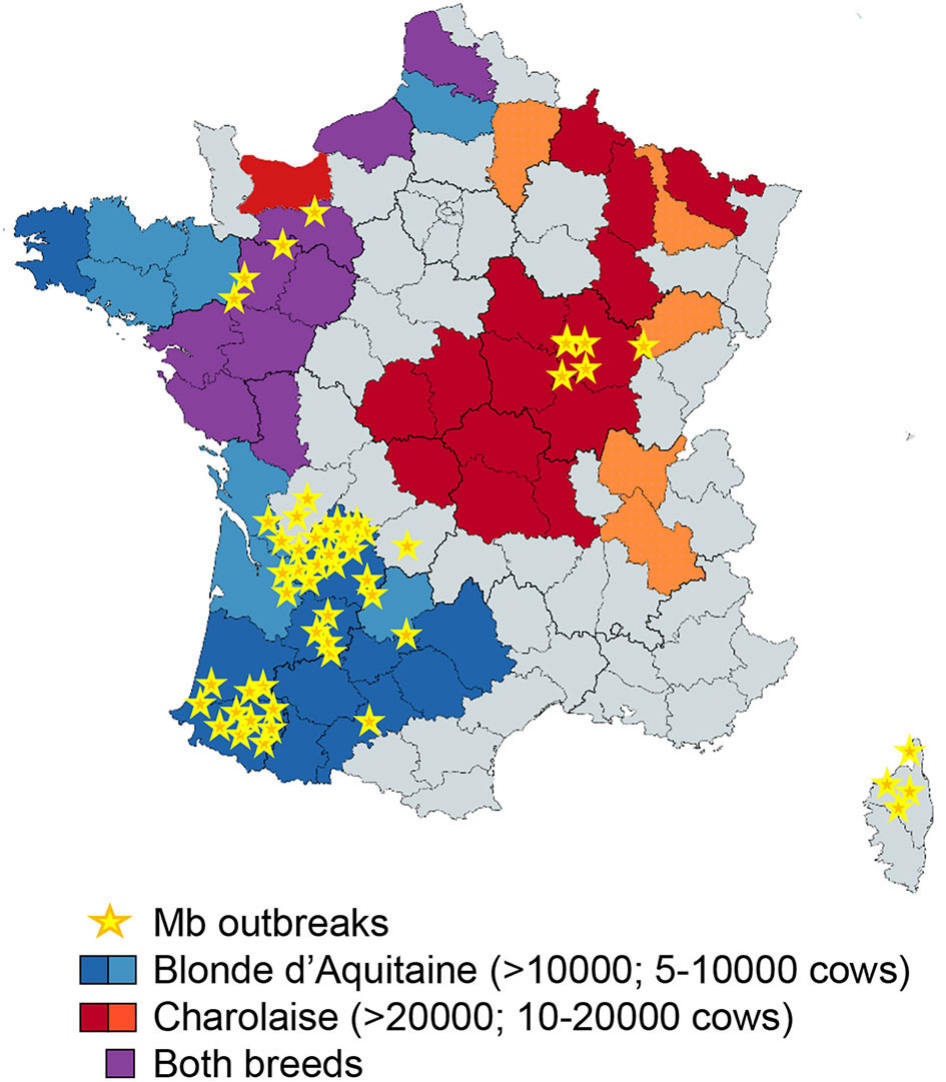
Superposition of Blonde d'Aquitaine and Charolaise beef breeds in French counties where Mb outbreaks were declared between December 2019 and 2020. This map of France shows the counties where Mb outbreaks were declared between December 2019 and December 2020 (yellow stars) and was obtained with data extracted from https://www.plateforme-esa.fr/. Herd densities of Blonde d'Aquitaine (blue), Charolaise (red), or both breeds (violet) were extracted from data obtained from https://www.racesdefrance.fr/ (cows above 3 years old have been considered).

Resistance to bTB is linked to the host genetics. Zebu breeds (*Bos indicus*) are more resistant to bTB disease than *Bos taurus*-derived breeds (49). Our results with PCLS, as a physiological model of the early lung response to infection, demonstrated striking differences between Blonde d'Aquitaine and Charolaise, emphasizing the importance of host genetics in response to Mb. It is not known whether the stronger inflammatory response of the Blonde d'Aquitaine tissue is associated with a greater sensitivity or resistance to Mb infection. While robust immunological responses are associated with an increased pathology at the level of the animal (31), at the cellular level, blood-derived MPs from animals with greater resistance to bTB (and that kill BCG more efficiently than cells from susceptible animals) produce higher levels of the pro-inflammatory mediators iNOS, IL-1β, TNFα, MIP1, and MIP3 (25). Although genetic selection of cattle would greatly complement bTB management and surveillance programs to control and ultimately eradicate the disease, especially in countries with the highest burden (50, 51), biomarkers to evaluate the resistance or susceptibility of cattle to Mb infection are critically missing. Some genomic regions and candidate genes have been identified in Holstein-Friesian cows, the most common dairy breed (52), and not surprisingly, these candidates are often involved in inflammation. A genomic region on chromosome 23, containing genes involved in the TNFα/NFκ-B signaling pathway, was strongly associated with host susceptibility to bTB infection (53). However, large within-breed analyses of Charolaise, Limousine, and Holstein-Friesian cattle identified 38 SNPs and 64 QTL regions associated with bTB susceptibility to infection (54). The genotyping of 1966 Holstein-Friesian dairy cows that were positive by skin test and either did or did not harbor visible bTB lesions, together with their skin test negative matched controls, led to the conclusion that these variable phenotypes following Mb exposure were governed by distinct and overlapping genetic variants (55). Thus, variation in the pathology of Mb seems to be controlled by a large number of loci and a combination of small effects. Similar conclusions were drawn from the genetic studies of human tuberculosis (56). In areas where Mb is highly prevalent, recurrent exposure to Mb may also imprint the bovine genome, and epigenetics could also contribute to the immune response in certain breeds. In France, the Nouvelle Aquitaine region accounted for 80% of Mb outbreaks last year. Interestingly, Blonde d'Aquitaine breed is very abundant in this area (Figure 7). Together with Limousine, another very abundant beef breed in this region, they contribute to most bTB outbreaks in Nouvelle Aquitaire (bovine tuberculosis national reference laboratory communication). In the future, comparisons between Blonde d'Aquitaine and Limousine would be interesting. In our study, Blonde d'Aquitaine or Charolaise cows were sampled from eight different French departments, none with recurrent Mb outbreaks, rendering previous exposure to Mb unlikely. Moreover, the breeding management was similar for the two breeds, as far as we could ascertain, suggesting that exposure to environment and possible wildlife sources would be comparable. We nevertheless observed striking differences in the early lung response to Mb infection between these two breeds, pointing to the possible control of Mb infection at the genetic or epigenetic level. Whether some cattle breeds are more susceptible to bTB than others remains an open question that deserves future studies with more consequent animal sampling. We furthermore believe that the PCLS model could greatly contribute to unraveling the role of tissue-level protective responses that would, in turn, reveal important biomarkers.

In addition to the cattle breed, our study pointed toward differences in the host response to distinct mycobacterial strains. The Mb strains were better inducers of a lung immune response than Mtb in cattle, which is in agreement with a previous work showing that Mtb H37Rv was attenuated *in vivo* in cattle compared to Mb AF2122 (13). *In vitro* studies with bovine AMPs infected with AF2122 or H37Rv revealed differences in the innate cytokine profiles: the CCL4, IL-1β, IL-6, and TNFα levels were more elevated in response to AF2122 than H37Rv (8), which is in agreement with our data. Interestingly, Mb3601, a representative strain of a highly successful genetic cluster that circulates both in cattle and wildlife in France (16), induced an inflammatory signature in the lung more efficiently than Mb AF2122. Whether this correlates with differences in Mb virulence in cattle or other mammals remains to be investigated; but, if this were the case, the PCLS model would be a practical tool to study and compare the virulence of Mb field strains compared to the *in vivo* experimental infection of cattle. Contrary to Mtb which is mostly restricted to humans, Mb is adapted to sustain across a large host range through repeated cycles of infection and transmission (57, 58). This remarkable trait is due to pathogen molecular genetic changes (59) that allow adapted bacilli to manipulate the host immune response to establish infection and disease and ultimately transmit infection to new, susceptible hosts (60, 61). We observed a weaker inflammation in the bovine lung after infection with Mtb compared to Mb, and it will be interesting to compare the ability of Mtb and Mb to induce inflammation in human PCLS obtained post-surgery. This latter comparative analysis could give clues on the links between lung innate inflammatory responses and host adaptation during TB.

Our most striking observation was the Mb-restricted induction of the type I IFN pathway in the bovine lung. This is in agreement with previous studies in bovine AMPs where cytosolic DNA-sensing pathways, in particular, RIG-I, were activated after 48 h of infection by Mb AF2122, but not Mtb H37Rv (32). In agreement with our data, these authors also demonstrated an induction of the RIG-I signaling pathway by Mb in AMPs (62). Therefore, AMPs contribute to type I IFN signaling in the lung. However, we also noticed differences between PCLS and AMPs in the induction of the IFN signature by Mb: for example, CXCL10 was detected in PCLS, but not in AMPs, in our study, which may be due to the time point used (63). However, it is also possible that other cells involved in crosstalk with AMPs contributed to CXCL10 production in response to Mb infection. Since CXCL10 has been proposed as a diagnostic biomarker of Mb infection in cattle (64), it will be interesting to better understand how this key mediator is regulated. Type I interferon favors Mb survival, and its induction may be a good manipulation strategy for the maintenance of infection. This manipulation mechanism, deciphered *in vitro* in murine bone marrow monocyte-derived MPs, involves the triggering of autophagy by cytosolic Mb DNA, in turn inducing IFNβ production. Autophagy antagonizes inflammasome activation to the benefit of Mb survival (65, 66). In C57BL/6 mice treated with IFNAR1 blocking Ab and infected with Mb, the recruitment of neutrophils was reduced, but the pro-inflammatory profile of MPs was increased, leading to a reduced bacillary burden (67). No impact on T-cells was observed in this *in vivo* model, revealing a role of type I IFN signaling during the innate phase of the host response to infection. Therefore, Mb exploits type I IFN signaling in many ways, and this pathway seems an important avenue to better understand Mb virulence. The PCLS model will greatly help to better dissect out this pathway in the lung during bTB. This could lead to new biomarkers to help genomic selection programs for cattle that are more resistant to bTB as well as new immunostimulation strategies counteracting the type I IFN pathway. This new knowledge will ultimately improve bTB control, a goal which is so greatly needed at the global level (68).

## Data Availability Statement

The original contributions presented in the study are included in the article/Supplementary Material, further inquiries can be directed to the corresponding author/s.

## Ethics Statement

Ethical review and approval was not required for the animal study because We only used post-mortem sampling at commercial abattoir.

## Author Contributions

AR designed and did most of the experiments, obtained funding, analyzed the data, prepared all the figures, and wrote the manuscript. FC performed experiments and prepared the inocula for experimental infections under BSL3 conditions. AC cultured AMPs and performed ELISA and q-RT-PCR. ED-D helped in PCLS experiments and revised the figures. MB provided the Mb3601 strain and revised the manuscript. AA provided the strain Mtb BTB1558. JB improved the RNA extraction protocol. DD and QM performed multiple experiments, and revised the manuscript. FA provided Ab and critically reviewed the imaging data. PG helped with transcriptomic analysis and revised the manuscript. SG obtained funding, designed the experiments, and revised the manuscript. NW obtained funding, supervised all aspects of the work, critically analyzed the data, and wrote the manuscript. All the authors read and approved the manuscript before publication.

## Conflict of Interest

The authors declare that the research was conducted in the absence of any commercial or financial relationships that could be construed as a potential conflict of interest. The handling editor declared a past collaboration with one of the authors SG.

## Supplementary Material

Supplementary Table 1 Sequences of primers used in this study. The primers were designed, using Geneious software, in intron-spanning regions when possible. The annealing temperature was set at 60°C. Housekeeping genes used as the reference to calculate ΔCT are indicated in the gray boxes.

Supplementary Figure 1 Age and geographical origin of the cows used in the study. The Charolaise and Blonde d'Aquitaine cows used were between 3 and 11 years old and came from eight different French departments. Two Blonde d'Aquitaine cows came from the same farm in Indre et Loire, and three Charolaise cows came from the same farm in Sarthe. All the other animals are from distinct farms. The data represent the age of individual animals and the median and interquartile range.

Supplementary Figure 2 Transcriptomic signature after infection with different doses of mycobacteria. Bovine precision-cut lung slices were obtained as described in Figure 1 and infected with 10^5^, 5 × 10^5^, 10^6^, or 5 × 10^6^ colony-forming units. The RNA was extracted 2 days post-infection, and *SDC4, CXCL1, HIF1*, and *OAS1* gene expressions were assessed with the Fluidigm Biomark. Individual data and the mean and standard deviation in each group are presented (*n* = 3 Charolaise). The dotted line represents the level of expression in the uninfected group.

Supplementary Figure 3 Cytokines/chemokines in precision-cut lung slice (PCLS) supernatants. The protein levels were measured in PCLS supernatant at 2 days post-infection with Multiplex. Individual data and the median and interquartile range in each group are presented (*n* = 6). ^*^*p* < 0.05 (Wilcoxon nonparametric test).

Supplementary Figure 4 Structure of the bovine precision-cut lung slices (PCLS) under a light microscope. The PCLS were observed under a light microscope (enlargement ×40 to ×200). The PCLS contain numerous alveoli and between one to three bronchioles, with thick and wavy epithelium that can be easily recognized (black asterisk, two views from the same area under two enlargements). Thin blood vessels (red dotted lines) were localized next to the bronchioles and diffused between the alveoli. No blood cells remained inside the endothelium (the cows were bled out at the abattoir). Alveolar macrophages can be seen inside the alveoli (black arrows).

Supplementary Figure 5 Localization of Mb3601-GFP in bovine precision-cut lung slices (PCLS). The PCLS were fixed at 2 days post-infection with 10^5^ colony-forming units of Mb3601-GFP recombinant strain and labeled with anti-pancytokeratine and anti-MHCII antibodies, which, respectively, revealed anti-pancytokeratine and Alexa 555 conjugated secondary Ab. The PCLS were transferred on cover slides and mounted with Fluoromount-G™ mounting medium containing DAPI. **(A)** The 3D images were analyzed with Leica LAS software. Z-stack imaging was performed at ×63 enlargement with a confocal microscope (10–15 μm in thickness, step size of 0.5–1 μm). Dotted white lines are drawn on the alveoli structure. **(B,C)** Crosshead sections illustrating Mb3601 inside **(B)** or near **(C)** an alveolar macrophage. X and Y projections are seen on the bottom and to the right of the picture; the intracellular localization of Mb3601-GFP is indicated by color merging (green + red = yellow). The results from one representative animal are shown (a total of *n* = 4 animals were analyzed).

Supplementary Video 1 Internalization of Mb3601 in alveolar macrophages after precision-cut lung slice (PCLS) *ex vivo* infection. The PCLS was fixed at 2 dpi with 10^5^ colony-forming units of Mb3601-GFP recombinant strain and labeled with anti-pancytokeratine and anti-MHCII antibodies, which, respectively, revealed anti-pancytokeratine and Alexa 555 conjugated secondary Ab. The PCLS was transferred on cover slides and mounted with Fluoromount-G™ mounting medium containing DAPI. Z-stack imaging was performed at ×63 enlargement with a confocal microscope. The 3D images were analyzed with Leica LAS software.
