# Supplementary material for: Mycobacterial Infection of Precision-Cut Lung Slices Reveals Type 1 Interferon Pathway Is Locally Induced by Mycobacterium bovis but Not M. tuberculosis in a Cattle Breed
Source: Front Vet Sci. 2021 Jul 9;8:696525. doi: 10.3389/fvets.2021.696525 (PMC8299756; doi:10.3389/fvets.2021.696525)
Supplement: Supplementary Table 1 — Sequences of primers used in this study. The primers were designed, using Geneious software, in intron-spanning regions when possible. The annealing temperature was set at 60°C. Housekeeping genes used as the reference to calculate ΔCT are indicated in the gray boxes. [file Data_Sheet_1.PDF]

Gene ID	Reference Sequences	Forward	Reverse
ACTB	NM_173979.3	ACGGGCAGGTCATCACCATC	AGCACCGTGTTGGCGTAGAG
C5aR1	NM_001007810	ATACCGTCCTTTGTGTTCCG	ATTGTAAGCGTGACCAGCG
CASP1	XM_024975697	CTCCCACCTGGCAGGAATAC	AGGAGCTGGAAAAGGAGGGA
CASP13	NM_176638.5	TCCGGACATTCAACAACCGT	ACCCACAATTCCCCACGATT
CASP8	NM_001045970.2	AATATTGGGGAGCAGCTGGG	AGGCATCCTTGATGGGTTCC
CCL2	NM_174006	GCTCGCTCAGCCAGATGCAA	GGACACTTGCTGCTGGTGACTC
CCL20	NM_174263	TTCGACTGCTGTCTCCGATA	GCACAACTTGTTTCACCCACT
CCL5	NM_175827	CTGCCTTCGCTGTCCTCCTGATG	TTCTCTGGGTTGGCGCACACCTG
CCR1	NM_001077839.1	ATGTCTTTGTCGGCGAGAGG	TCTGTGGACAGGAAGGGGAA
CD14	NM_174008	TCCACAGTCCAGCCGACAAC	AACGGCGCTAGACCAGTCAG
CD209	NM_001145756.1	CACCCTCGACCACTACACAG	TGAAGAAGCCCAGTGAGACG
CD83	NM_001046590.1	GAAGGGCAGAGAAACCTGAC	AGAGGTGACTGGGAGGAAAG
CLEC4A	NM_001191510.1	GAAGTTACCACCGTGCTTGC	CCTCTGAAGTCATGCTGCGA
CLEC6A	NM_001034479.1	TACCTGGAAGCCGTTTGGTT	CAAGTGAGCTCCCATCCCAA
CLEC7A	NM_001031852.1	AGGCAAGTGTCTTCCCAGC	ACAACAAGGTGGAGCCATCC
CX3CR1	NM_001102558	TGGCCTTGGGAACTGTCTTC	TGGCTGTTGATGAGGGCAAA
CXCL1	NM_175700.2	CCAAACCGAAGTCATAGCC	TCAGTTGGCACTAGCCTTGTTTAGC
CXCL10	NM_001046551	TTCAGGCAGTCTGAGCCTAC	ACGTGGGCAGGATTGACTTG
CXCL2	NM_174299.3	GTGTCTCAACCCCGCCGCTC	TCCAGATGGCCTTAGGAGGTGG
CXCL3	NM_001046513.2	AGCGATGCTGCTCCTGCTCCT	CCATGGGAGCTTCAGGGTTGAG
CXCL5	NM_174300.2	TGTTTAACCACCACACCGGG	AGGTGGCTATCACTTCCACC
CXCL8	NM_173925.2	TGAAGCTGCAGTTCTGTCAAG	TTCTGCACCCACTTTTCCTTGG
CXCR1	NM_174360	ACATGGTTGGTGACTCAGTCTT	CGTGCCGCTGTAATTTCCAA
CXCR2	NM_001101285	ACAGGTGACAAGCCCAGAATC	CGACCAATCCGGCTGTATAA
CXCR3	NM_001011673	CCACAGGACTTCAGCCTCAA	CGACTGCCACGATGCCATTA
DEFB5	NM_001130761	TCGTGCTCCTCTTCCTAGTC	GGCACGAGATCGGAATACAG
GAPDH	NM_001034034.2	GGCATCGTGGAGGGACTTATG	GCCAGTGAGCTTCCCGTTGAG
HIF1	NM_174339	ACCCTGCACTCAACCAAGAA	TGGGACTGTTAGGCTCAGGT
IFITM3	NM_001078141	CCTGAACATCTGCTCCCTGG	CTCGGAGACTGCTTGAACGA
IFNAR1	NM_174552.2	TCCTTTGCCACGTGTCAAGT	AGTAGCGTGAGGGAGACAGA
IFNB	XM_005209900	GCTACAGCTTGCTTCGATTC	TGTGCTGGAGCATCTCATAC
IFN-γ	NM_174086	ACCAGGTCATTCAAAGGAGCAT	TCTGCAGATCATCCACCGGA
IL10	NM_174088.1	GTGATGCCACAGGCTGAGAA	TGCTCTTGTTTTCGCAGGGCAG
IL12p35	NM_174355	ACAGAAGGCCAGACAAACTC	AGCCAGACAATGCCCATTAG
IL12p40	NM_174356	CACCAGCAGCTTCTTCATCA	CTTGTGGCATGTGACTTTGG
IL13	NM_174089.1	CATGGCGCTCTTATTGACCG	AATGAGCTCCTTGAGGGCTG
IL15	NM_174090	AACAGCGATGCAGTGCTTTC	TCCTCCAGTTCCTCACATTC
IL17A	NM_001008412	GCCCACCTACTGAGGACAAG	GCTGGATGGTGACAGAGTTC
IL17C	617538	TGACGTCCACCAGCGCTCCATC	CTGGACCAGCGGCACTGAGTTG
IL17F	NM_001192082	CACTCTGGAGGACCACATTG	GAGTTCAGGGTCCTGTCTTC
IL17RA	XM_024992765	GGCTGAACTGCACAGTCAAG	AGCGTCCACTCGATGTGAAC
IL17RB	NM_001083467.1	GTCCCTCCATGGCTGTGAAC	AGCGCCATGTATCTGTCTCC
IL17RC	NM_001075178	TGCCCTGGTTCCTTCTGTCC	AGGCAGAGCACGTCACCATC
IL17RE	XM_010817592.3	CTGGGAGCCACACTGTAGAC	GTCACGGCCATGACCATCTG
IL1-alpha	NM_174092	CTGAAGAAGAGACGGTTGAG	ATGCATTCCTGGTGGATGAC
IL1-beta	NM_174093.1	CTCTCACAGGAAATGAACCGAG	GCTGCAGGGTGGGCGTATCACC
IL21	NM_198832	GTGGCCCATAAGTCAAGCTC	CGCTCACAGTGTCTCTTTAC
IL22	NM_001098379	AGGAGCCCTACATCTTCAAC	CTTCGTCACCTGATGGATTC
IL23p19	NM_001205688	GATGGCTGTGATCCACAAGG	TGGGAATAGGGCTTGGAGTC
IL26	NM_001205424	CAGAGCAACGATTCCAGAAG	TCTGCCTGAGGCTATGAAAG
IL33	NM_001075297.1	GATGGTGGCAGTCATCGGAA	GTAGCTCCACAGAGTGCTCC
IL4	NM_173921.2	GCCACACGTGCTTGAACAAA	CTTGTGCTCGTCTTGGCTTC
IL5	NM_173922.1	CAAACTGCACAAGGGGATGC	ATCTTTCTCCTCCACACTTCCT
IL6	NM_173923.2	TGCTGGTCTTCTGGAGTATC	GTGGCTGGAGTGGTTATTAG
IRF3	NM_001029845.3	GGAAGGATAAGCCCGACCTG	GAGTCCTTGCTGTGGTCCTC

Gene ID	Reference Sequences	Forward	Reverse
IRF7	NM_001105040.1	AAGTCTACTGGGAGGTGGGG	CCGAAGTCAAAGATGGGCGT
ISG15	NM_174366	CGCCCAGAAGATCAATGTGC	TCCTCACCAGGATGGAGATG
ITGAM	NM_001039957.1	TTGAGGCGACGATGGAGTTC	ACTTTCACCTGCCCAGCAAT
LAP	NM_203435	TGCTCCTTGCGCTCCTCTTC	CTCCGAGACAGGTGCCAATC
LGP2	NM_001015545.1	CCCTTCACTGTGCCTGACTT	AGGTTGTACTGGGCATTGCA
MD2	NM_001046517.1	AATCGTTGGGTCTGCAACTC	GCGCAATGGGAAATTCATGG
MIF	NM_001033608	GCAAGCCGGCACAGTACATC	CCGCGTTCATGTCGCAGAAG
MMP2	NM_174745	CCAAGGGTACAGCCTGTTCC	GGCCGGTGCCAGTATCAATG
MMP9	NM_174744	CGTTCCGACGACATGCTCTG	CATTGCCGTCCTGGGTGTAG
MUC1	NM_174115	CTCTCCAGGCCATGATAGTG	AAGTGACCATGGAGCTTGAC
MX1	NM_173940.2	GGCCACATCCCCTTGATCAT	CGTACTGGTCCTTGTCCTGG
NLRP3	NM_001102219.1	CTCAGTGGCAATACCCTGGG	AGCACTGTCCCAACCACAAT
NOD1	NM_001256563.1	TGGTCACTCACATCCGAAAC	AGGCCTGAGATCCACATAAG
NOD2	NM_001002889	CCCAGGGGCTCAGAACTAACA	CCTTCATCCTGGACGTGGTTC
NOS2	NM_001076799	CTTGAGCGAGTGGTGGATGG	ATCTGAGGGCTGGCATAGGG
OAS1Z	NM_001029846.2	CCAATGGTTCTTCTGCCCCT	GGCAGGAGGTGGTCTTTGAT
PKR	NM_178109.3	TTTTCGCCTCCTCCTCATGC	AACGAATACAGGCTCGCAGA
PPIA	NM_178320.2	TCCGGGATTTATGTGCCAGGG	GCTTGCCATCCAACCACTCAG
PTGS2	NM_174445.2	CATGGGTGTGAAAGGGAGGAA	ATTTGTGCCCTGGGGATCAG
PTX3	NM_001076259	TGCCTGCATTTGGGTCAAAG	CACGTTCCTAGGGAAATCAC
RIG-I	XM_002689480	TGTGGTGGAAGATGTTGCGA	AGGGGACATTTCTGCAGCAT
S100A7	NM_174596	CAGCTTGAGCAGGCCATTAC	CGTGGCTGTGGTTGTGATAG
S100A8	NM_001113725	CTCCCTGATTGACGTCTACC	TCCAGGCCCACCTTTATCAC
S100A9	NM_001046328	TGACACCCTGATCCAGAAAG	GCCACCAGCATAATGAACTC
SAA3	NM_181016	CCTCAAGGAAGCTGGTCAAG	TACCTGGTCCCTGGTCATAC
SDC4	XM_025001268	AGCTTCAGACAGGGCCTTTC	GTCATGAGCGGGGAAGTAGG
STAT1	NM_001077900.1	CAAAGGAAGCCCCAGAGCCTAT	GCCACTCTTCTGTGTTCACTTAC
TAP	NM_174776.1	GTAGGAAATCCTGTAAGCTGTG	GTGTCTTGGCCTTCTTTTAC
TGFB1	XM_024977949	CCTGAGCCAGAGGCGGACTAC	GCTCGGACGTGTTGAAGAAC
TLR1	NM_001046504.1	ACCCTACTCTGAACCTCAAG	GACTGCACACTGGATTTCTG
TLR2	NM_174197.2	ACTGGGTGGAGAACCTCATGGTCC	ATCTTCCGCAGCTTACAGAAGC
TLR3	NM_001008664.1	TTTGCCTGGCTTCCACATCT	GGCGTCTCAAGTTGGAAAGC
TLR4	NM_174198.6	GCATGGAGCTGAATCTCTAC	CAGGCTAAACTCTGGATAGG
TLR5	NM_001040501.1	TTCCTGCAACCTCACCCAAG	CTGAGATTGGGCAGGTTTCG
TLR6	NM_001001159	CTCCGGGAGATAGTCACTTC	GGCCCTGGATTCTATTATGG
TLR7	NM_001033761.1	GCATCTCTCCAGCCTCCTTT	CACACGTTGTCTTTTGGCCC
TLR8	NM_001033937.1	AATGCCAAGTCCCAGAGTGG	CCAGCAGCAACTCCCTTAGG
TLR9	NM_183081.1	GACCTGTCCCACAACAAGCT	TGAAGGGCTGGCTGTTGTAG
TNF alpha	NM_173966.3	TCTTCTCAAGCCTCAAGTAACAAGC	CCATGAGGGCATTGGCATAC
TSLP	XM_024995349	AGAGAGCTACCGGAACATCA	GGGCTGGTCTTCACAGTAGA
