# Supplementary figures and images for: Mycobacterial Infection of Precision-Cut Lung Slices Reveals Type 1 Interferon Pathway Is Locally Induced by Mycobacterium bovis but Not M. tuberculosis in a Cattle Breed
Source: Front Vet Sci. 2021 Jul 9;8:696525. doi: 10.3389/fvets.2021.696525 (PMC8299756; doi:10.3389/fvets.2021.696525)

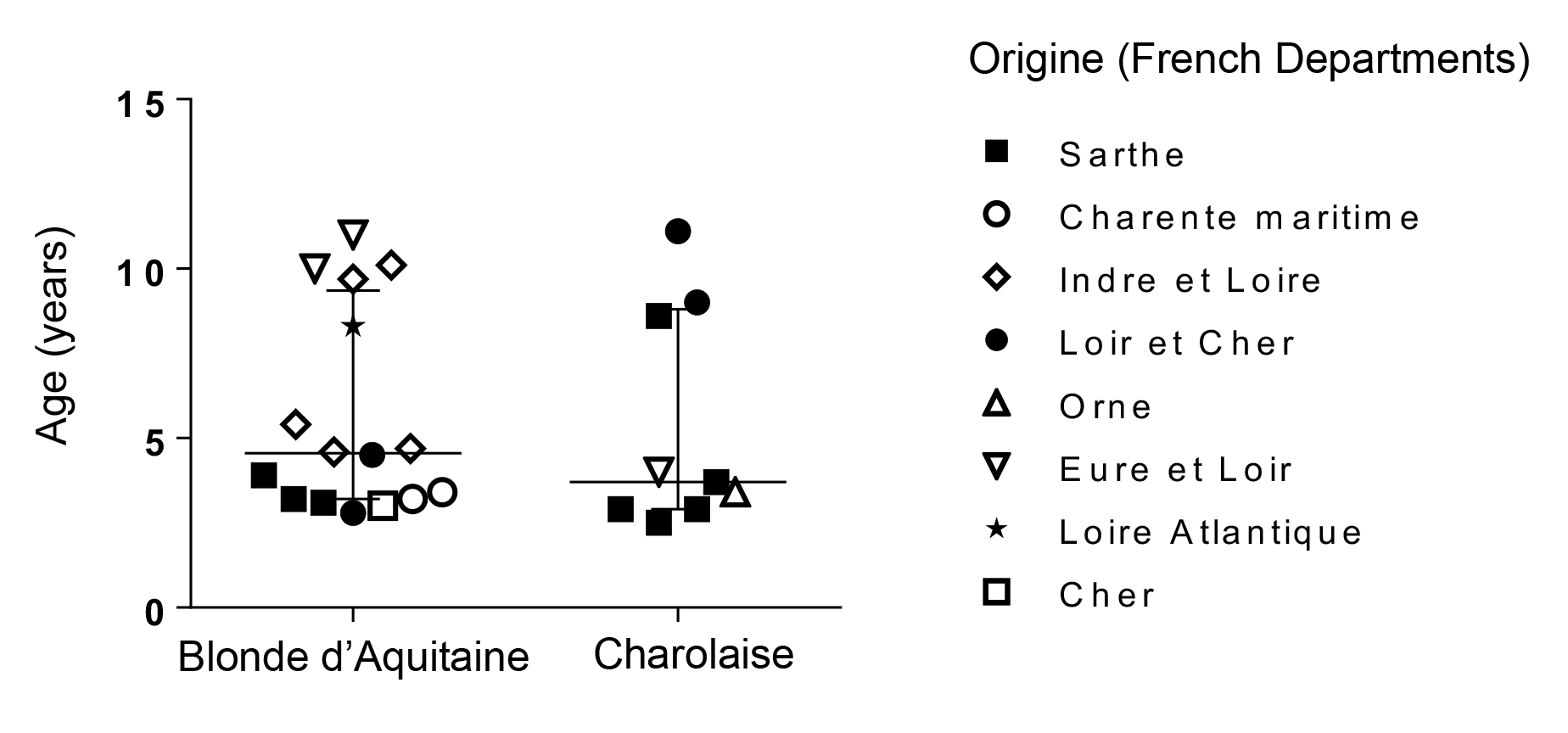

Supplement: Supplementary Figure 1 — Age and geographical origin of the cows used in the study. The Charolaise and Blonde d'Aquitaine cows used were between 3 and 11 years old and came from eight different French departments. Two Blonde d'Aquitaine cows came from the same farm in Indre et Loire, and three Charolaise cows came from the same farm in Sarthe. All the other animals are from distinct farms. The data represent the age of individual animals and the median and interquartile range. [file Image_1.TIF]

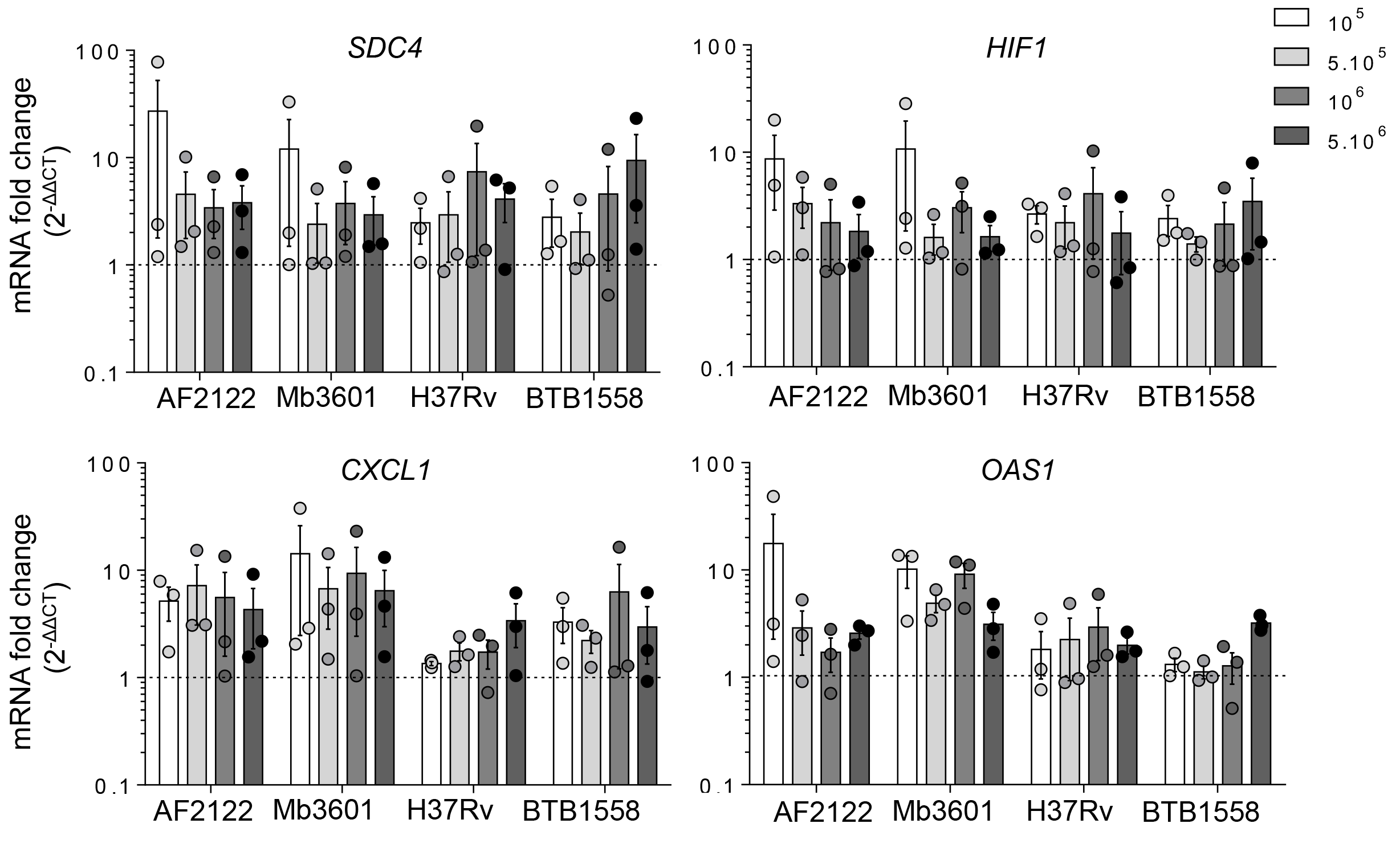

Supplement: Supplementary Figure 2 — Transcriptomic signature after infection with different doses of mycobacteria. Bovine precision-cut lung slices were obtained as described in Figure 1 and infected with 105, 5 × 10^5^, 106, or 5 × 10^6^ colony-forming units. The RNA was extracted 2 days post-infection, and SDC4, CXCL1, HIF1, and OAS1 gene expressions were assessed with the Fluidigm Biomark. Individual data and the mean and standard deviation in each group are presented (n = 3 Charolaise). The dotted line represents the level of expression in the uninfected group. [file Image_2.TIF]

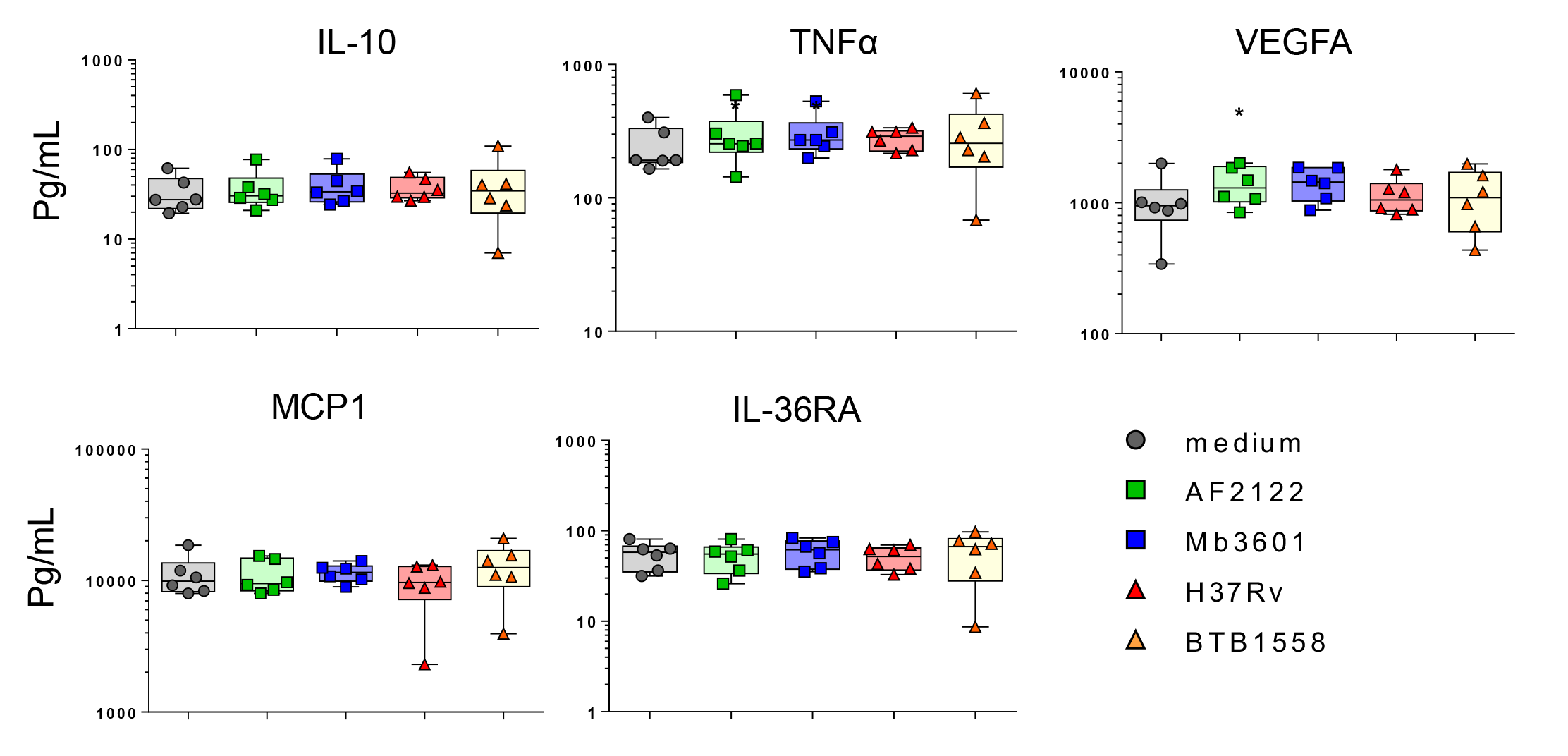

Supplement: Supplementary Figure 3 — Cytokines/chemokines in precision-cut lung slice (PCLS) supernatants. The protein levels were measured in PCLS supernatant at 2 days post-infection with Multiplex. Individual data and the median and interquartile range in each group are presented (n = 6). *p < 0.05 (Wilcoxon nonparametric test). [file Image_3.TIF]

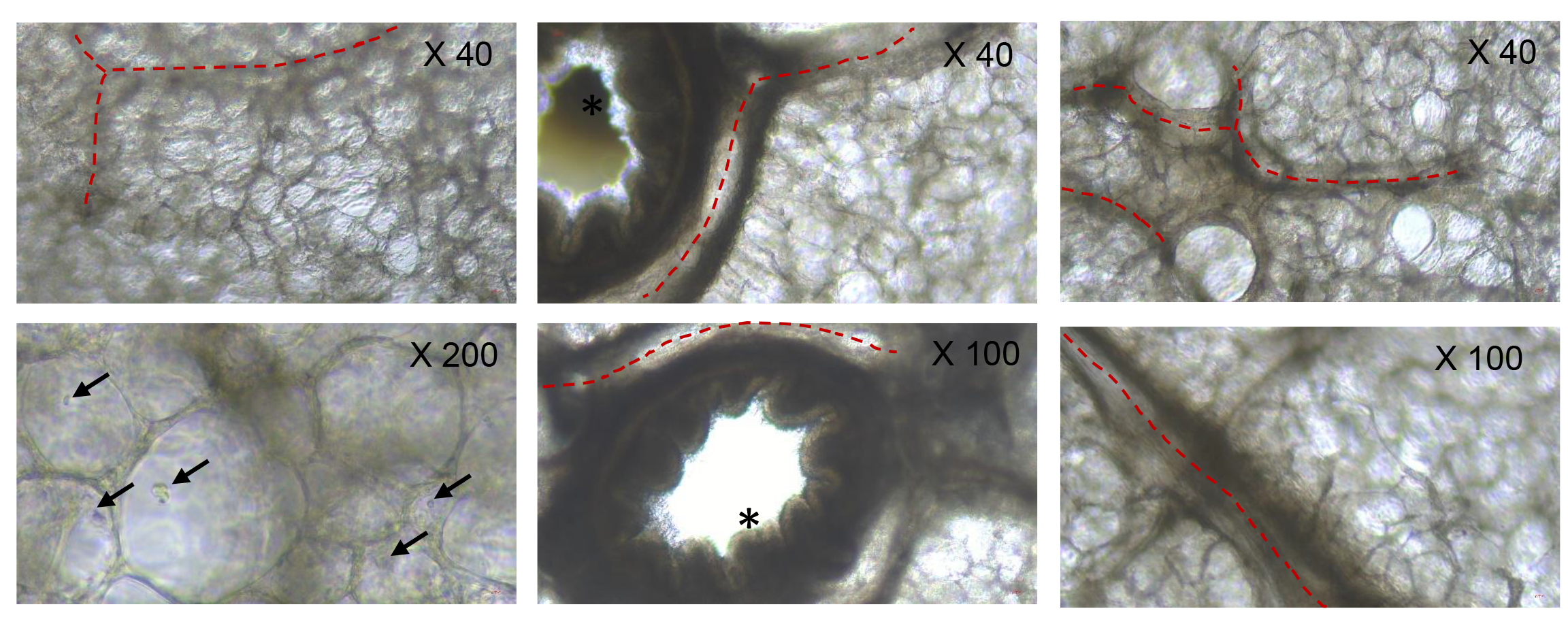

Supplement: Supplementary Figure 4 — Structure of the bovine precision-cut lung slices (PCLS) under a light microscope. The PCLS were observed under a light microscope (enlargement ×40 to ×200). The PCLS contain numerous alveoli and between one to three bronchioles, with thick and wavy epithelium that can be easily recognized (black asterisk, two views from the same area under two enlargements). Thin blood vessels (red dotted lines) were localized next to the bronchioles and diffused between the alveoli. No blood cells remained inside the endothelium (the cows were bled out at the abattoir). Alveolar macrophages can be seen inside the alveoli (black arrows). [file Image_4.TIF]

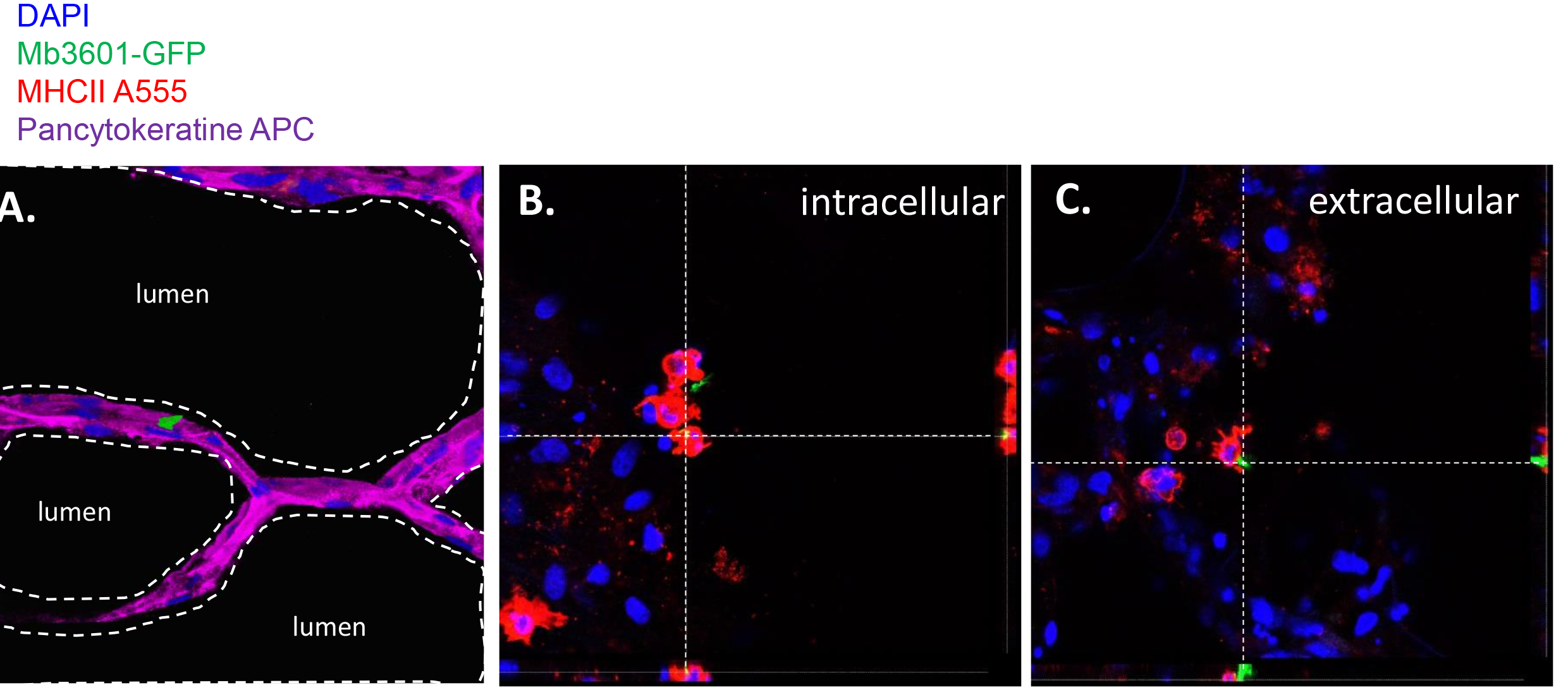

Supplement: Supplementary Figure 5 — Localization of Mb3601-GFP in bovine precision-cut lung slices (PCLS). The PCLS were fixed at 2 days post-infection with 105 colony-forming units of Mb3601-GFP recombinant strain and labeled with anti-pancytokeratine and anti-MHCII antibodies, which, respectively, revealed anti-pancytokeratine and Alexa 555 conjugated secondary Ab. The PCLS were transferred on cover slides and mounted with Fluoromount-G™ mounting medium containing DAPI. (A) The 3D images were analyzed with Leica LAS software. Z-stack imaging was performed at ×63 enlargement with a confocal microscope (10–15 μm in thickness, step size of 0.5–1 μm). Dotted white lines are drawn on the alveoli structure. (B,C) Crosshead sections illustrating Mb3601 inside (B) or near (C) an alveolar macrophage. X and Y projections are seen on the bottom and to the right of the picture; the intracellular localization of Mb3601-GFP is indicated by color merging (green + red = yellow). The results from one representative animal are shown (a total of n = 4 animals were analyzed). [file Image_5.TIF]
